# Causal effects of gut microbiota on sepsis and sepsis-related death: insights from genome-wide Mendelian randomization, single-cell RNA, bulk RNA sequencing, and network pharmacology

**DOI:** 10.1186/s12967-023-04835-8

**Published:** 2024-01-02

**Authors:** Sha Yang, Jing Guo, Zhuo Kong, Mei Deng, Jingjing Da, Xin Lin, Shuo Peng, Junwu Fu, Tao Luo, Jun Ma, Hao Yin, Lin Liu, Jian Liu, Yan Zha, Ying Tan, Jiqin Zhang

**Affiliations:** 1 Guizhou University Medical College, Guiyang, 550025 Guizhou China; 2 Department of Anesthesiology, Guizhou Provincial People’s Hospital, Guiyang, China; 3 Department of Neurosurgery, Guizhou Provincial People’s Hospital, Guiyang, China; 4 Department of Nephrology, Guizhou Provincial People’s Hospital, Guiyang, China; 5 Department of Respiratory and Critical Care Medicine, Guizhou Provincial People’s Hospital, Guiyang, China

**Keywords:** Mendelian randomization, Sepsis, Gut microbiota, Single-cell RNA-seq, Network pharmacology, Transcriptomics

## Abstract

**Background:**

Gut microbiota alterations have been implicated in sepsis and related infectious diseases, but the causal relationship and underlying mechanisms remain unclear.

**Methods:**

We evaluated the association between gut microbiota composition and sepsis using two-sample Mendelian randomization (MR) analysis based on published genome-wide association study (GWAS) summary statistics. Sensitivity analyses were conducted to validate the robustness of the results. Reverse MR analysis and integration of GWAS and expression quantitative trait loci (eQTL) data were performed to identify potential genes and therapeutic targets.

**Results:**

Our analysis identified 11 causal bacterial taxa associated with sepsis, with increased abundance of six taxa showing positive causal relationships. Ten taxa had causal effects on the 28-day survival outcome of septic patients, with increased abundance of six taxa showing positive associations. Sensitivity analyses confirmed the robustness of these associations. Reverse MR analysis did not provide evidence of reverse causality. Integration of GWAS and eQTL data revealed 76 genes passing the summary data-based Mendelian randomization (SMR) test. Differential expression of these genes was observed between sepsis patients and healthy individuals. These genes represent potential therapeutic targets for sepsis. Molecular docking analysis predicted potential drug-target interactions, further supporting their therapeutic potential.

**Conclusion:**

Our study provides insights for the development of personalized treatment strategies for sepsis and offers preliminary candidate targets and drugs for future drug development.

**Supplementary Information:**

The online version contains supplementary material available at 10.1186/s12967-023-04835-8.

## Introduction

Sepsis is a severe infectious disease that exhibits a rising incidence and mortality rate globally, posing a significant challenge in the field of public health. Epidemiological data indicates that sepsis affects millions of people annually, with a mortality rate ranging from 30 to 50% [1, 2]. The development of sepsis is complex and rapid, often accompanied by severe inflammatory responses and multiple organ dysfunction syndrome (MODS), imposing substantial pathological and physiological burdens and posing a threat to patients’ lives [3]. Despite certain advancements in sepsis treatment, such as early administration of antibiotics, and supportive care, the mortality rate remains high, and treatment outcomes are still suboptimal [4]. Therefore, it is imperative to gain a deep understanding of the pathological mechanisms underlying sepsis and explore novel therapeutic approaches.

The dysbiosis of the gut microbiota has been closely associated with the occurrence and progression of various diseases, including sepsis [5–7]. When the gut microbiota loses its balance, there is a decrease in beneficial microbial populations and an increase in harmful microbial populations. This imbalance leads to the proliferation of detrimental microbes and disrupts the integrity of the intestinal barrier. Consequently, pathogens and toxins can traverse the compromised intestinal barrier and enter the circulatory system, triggering an immune inflammatory response [6]. This immune inflammatory response may be a key factor in the occurrence and progression of sepsis. Several studies have confirmed the relationship between the gut microbiota and sepsis [8–17]. The study revealed significant differences in gut microbiota between sepsis patients and healthy individuals [9]. During sepsis onset, the dysbiosis of gut microbiota is closely associated with the severity of infection and inflammatory response [10, 11]. Furthermore, some studies have found that specific harmful bacterial groups in the gut microbiota, such as Enterococcus and Escherichia coli, may be associated with the occurrence and worsening of sepsis. Investigating the relationship between gut microbiota and sepsis contributes to a better understanding of the pathogenesis of this disease [18]. The gut microbiota plays a crucial role in the occurrence and development of sepsis by modulating host immune function and influencing intestinal barrier integrity. However, there are still many unknowns regarding the specific mechanisms and influencing factors. Further research is needed to explore the balance between beneficial and harmful bacterial groups and the molecular mechanisms by which the microbiota regulates immune and inflammatory responses.

However, there are some limitations in the current research on the relationship between sepsis and the gut microbiota. Firstly, our understanding of the composition and function of the gut microbiota remains limited, and the underlying mechanisms of different microbial populations and their association with sepsis have not been fully elucidated. Secondly, the lack of large-scale, multicenter clinical research data has resulted in an incomplete and inaccurate understanding of the relationship between the gut microbiota and sepsis in different patient populations. Furthermore, due to limitations in research methods, the causal relationship between the gut microbiota and sepsis, as well as its potential applications in sepsis prevention and treatment, have not been extensively investigated.

We will employ Mendelian randomization (MR) study design, which is a powerful epidemiological tool for causal inference [19]. In contrast to traditional observational studies, MR utilizes genetic variations as instrumental variables that are naturally randomized, enabling the assessment of causal relationships between the gut microbiota and sepsis [20]. This approach will help determine the true role of the gut microbiota in the occurrence and development of sepsis. Furthermore, this study will integrate single-cell transcriptomics and bulk RNA sequencing technologies to comprehensively elucidate the underlying mechanisms of the gut microbiota in sepsis development [21, 22]. Single-cell transcriptomics provides high-resolution cellular types and functional characteristics, aiding in a better understanding of the interplay between the gut microbiota and sepsis [23]. Meanwhile, bulk RNA sequencing offers overall gene expression information to further validate and complement the results obtained from single-cell transcriptomics. In addition, our research will analyze and dock potential therapeutic drugs to explore novel treatment strategies for sepsis [24]. By combining the regulatory mechanisms of the gut microbiota and existing drug databases, we can identify potential therapeutic agents and further verify their effectiveness and safety. We aim to provide a more accurate assessment of the relationship between the gut microbiota and sepsis, reveal its mechanisms of action, and provide new clues and strategies for personalized treatment of sepsis.

## Methods

### Study design

**Fig. 1:**
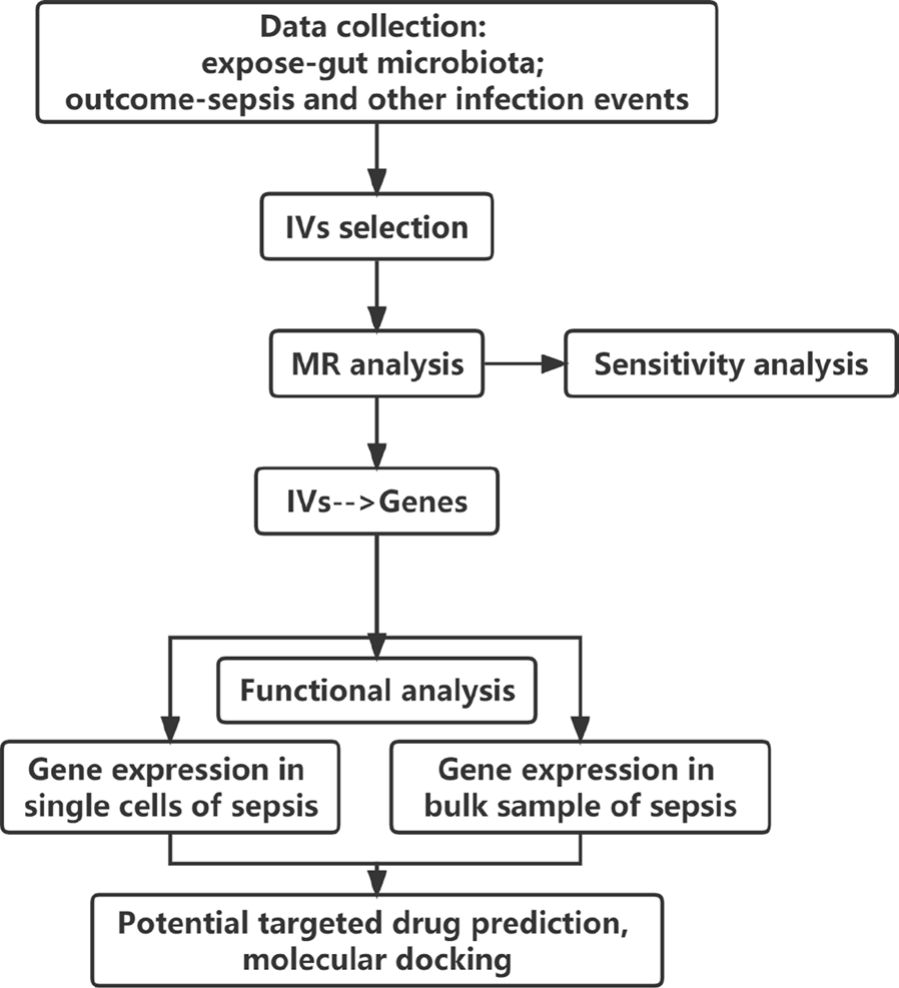
The flowchart of the study

This study followed the STROBE-MR guidelines [25] and adhered to the key principles of the Strengthening the Reporting of Observational Studies in Epidemiology (STROBE) guidelines [26]. The MR method relies on three assumptions [27]: (1) The genetic variants serving as instrumental variables are associated with specific gut microbiota taxa, including 211 taxa, 131 genera, 35 families, 20 orders, 16 classes, and 9 phyla; (2) The genetic variants are unrelated to any unmeasured confounding factors associated with sepsis and other infections; (3) The genetic variants are exclusively associated with sepsis and other infection events through the gut microbiota taxa, rather than through other pathways. Our analysis utilized publicly available GWAS summary statistics. No new data were collected, and no additional ethical approval was required. The study research process is illustrated in Fig. 1. Finally, reverse MR analysis was conducted to mitigate the potential impact of sepsis and other infection events on the gut microbiota.

### Data sources

The genetic data of the gut microbiota used in this study were obtained from the latest genome-wide association study (GWAS) summary data conducted by the Mibiogen Consortium. The data included 18,340 participants from 24 cohorts, with 85% of European ancestry. Based on the 16S rRNA gene sequencing results, we assessed 211 taxonomic groups at six hierarchical levels, including 9 phyla, 16 classes, 20 orders, 35 families, and 131 genera [28]. For the primary outcome, we obtained sepsis data from the UK Biobank, a large-scale population-based cohort study of UK adults, as previously reported [29]. The dataset included 11,643 cases of sepsis and 474,841 controls of European ancestry [30]. For the secondary outcomes, we obtained sepsis survival data, COPD/ asthma/ ILD-related pneumonia or pneumonia-derived septicaemia, COPD/ asthma-related pneumonia or pneumonia-derived septicaemia, and asthma-related pneumonia or sepsis. To acquire a larger sample of sepsis survival data, we utilized summary data from previous genome-wide association studies (GWAS) on sepsis survival. This study included data from four cohorts: GenOSept (Genetics of Sepsis and Septic Shock in Europe) consortium, GAinS (Genomic Advances in Sepsis) study, VASST (Vasopressin in Septic Shock Trial), and PROWESS (Protein C Worldwide Evaluation in Severe Sepsis) trial [30]. Among these cohorts, 1896 patients died within 28 days of admission, and there were 484,588 controls of European ancestry [31]. Data for other secondary outcomes were derived from a large prospective cohort study in Finland, FinnGen (Round 10), conducted in collaboration with multiple institutions and linked to electronic health record data [32]. Each cohort included 27,715 cases and 159,867 controls, 27,715 cases and 159,867 controls, and 5545 cases and 135,449 controls of European ancestry, respectively.

The selection of instrumental variables for bidirectional Mendelian randomization (MR) followed the following criteria. (1) A significance threshold for each microbial taxonomic group within the locus range was set at p < 1.0 × 10^–5^ [33]. (2) The 1000 Genomes European reference panel was used to calculate linkage disequilibrium (LD) between single nucleotide polymorphisms (SNPs), and SNPs with an LD threshold of r2 < 0.01 were retained, prioritizing SNPs with lower p-values. (3) Only SNPs with an effect allele frequency (EAF) > 0.01 were retained. (4) Palindromic SNPs were removed. (5) SNPs with an F-statistic < 10 were excluded to avoid weak instrumental variable bias.

### MR statistical analysis

We employed the inverse variance-weighted (IVW) method as the primary approach, along with MR Egger, weighted median, simple mode, and weighted mode, for MR analysis to assess the causal effects of gut microbiota on sepsis and other infection-related events [34–38]. The Wald ratio method was used to estimate the effects of each SNP. Cochrane’s Q test was applied to assess heterogeneity among the SNP instruments. In the presence of heterogeneity (p < 0.05), the random-effects IVW test was conducted for conservative but robust estimation. The weighted median test can yield consistent estimates when ≥ 50% of the weights come from valid IVs. The MR-Egger regression test allows for the presence of pleiotropy in more than 50% of the IVs. We selected MR-Egger intercept test, global test for outliers (MR-PRESSO), and leave-one-out analysis as sensitivity analysis methods. The MR-Egger intercept, Mendelian randomization pleiotropy residual sum and outlier (MR-PRESSO) global test were used to detect the degree of pleiotropy [39]. Leave-one-out analysis assessed whether significant results were driven by individual SNPs [40].

All statistical analyses were performed using R (version 4.1.3). The IVW, weighted median, simple mode, weighted mode, and MR Egger regression methods were implemented using the “TwoSampleMR” package (version 0.5.4) [41]. The MR-PRESSO test was conducted using the “MRPRESSO” package. The significance threshold was set at a p-value < 0.05.

### Mapping SNPs to genes

We utilized the online database SNPnexus (https://www.snp-nexus.org/v4/), a web-based variant annotation tool, to map each queried variant to its closest gene, which could be an overlapped gene or a downstream or upstream gene [42]. Subsequently, based on the results mentioned above, we conducted MR analysis on the IV SNPs and the exposure genes. The overall level statistics for eQTLs were derived from the CAGE study [43], which investigated gene expression at the transcript level in peripheral blood from 2765 individuals, primarily of European descent. All independent eQTLs for the focal gene with a conditional p-value < 0.05 were included for further analysis.

### Functional enrichment analysis of key genes

We considered an odds ratio (OR) greater than 1 as indicating harmful gut microbiota, while an OR less than 1 indicated beneficial gut microbiota in our MR analysis. Functional enrichment analysis was performed separately on the genes mapped by the IVs for the two groups of gut microbiota. The “clusterProfiler” R package [44] was employed for enrichment analysis of Gene Ontology (GO) Biological Processes (BP), Cellular Components (CC), Molecular Functions (MF), and KEGG pathways. We set the significance threshold at p < 0.05, and the top 10 most significant GO terms and pathways were visualized using the “ggplot2” R package.

### Single-cell RNA sequencing data analysis

We obtained the single-cell RNA-sequencing (scRNA-seq) data of sepsis patients (GSE167363) from the GEO database, which included human peripheral blood mononuclear cells from 2 healthy controls, 4 survivors, and 6 non-survivors of gram-negative sepsis patients [45]. The publicly available dataset used in this study had obtained the necessary ethical approvals. The “Seurat” R package was utilized for the analysis of scRNA-seq data [46]. After filtering low-quality data, which included genes expressed in fewer than 3 single cells, cells expressing fewer than 1000 genes, and cells with mitochondrial gene content exceeding 20%, we proceeded with further analysis. Subsequently, we employed the “NormalizeData” function for “LogNormalize” normalization of the data, followed by conversion into a Seurat object. The “FindVariableFeatures” function was used to identify the top 2500 highly variable genes. Next, the “RunPCA” function was used for principal component analysis (PCA) of the highly variable genes, selecting the top 15 principal components (PCs). Cell clustering analysis was performed using the “FindNeighbors” and “FindClusters” functions. Uniform Manifold Approximation and Projection (UMAP) was then conducted using the “RunUMAP” function, and cell clustering experiments were performed based on UMAP-1 and UMAP-2. To annotate the cell clusters with cell types, we utilized the “SingleR” R package and performed cell annotation using the Human Primary Cell Atlas as the reference dataset [47]. Finally, the expression patterns of the aforementioned genes in various cell types were visualized based on the UMAP plot and bubble plot.

### Bulk RNA data analysis

We downloaded the data GSE65682 from the Gene Expression Omnibus (GEO) database database (https://www.ncbi.nlm.nih.gov/geo/) [48]. This dataset provides reliable expression profiles of sepsis, exclusively derived from human samples. It includes 760 sepsis blood samples and 42 healthy control blood samples, consisting of 365 samples from patients who survived for 28 days and 114 samples from patients who died within 28 days. The data was generated using the GPL13667 [HG-U219] Affymetrix Human Genome U219 Array platform. After standardizing, annotating, and cleaning the clinical information of the GSE65682 dataset, I used the limma R package to identify the Differentially Expressed Genes (DEGs) between the sepsis and healthy control groups, as well as between the samples from patients who died within 28 days and those who survived for 28 days. The criteria for identifying DEGs were set to a p-value < 0.05.

### Potential therapeutic drugs prediction

We searched each key gene on the CTDbase database to obtain information on drug interactions and/or diseases associated with these genes. Subsequently, we analyzed small molecule ligands that act on these genes in sepsis. For protective genes, we identified drugs that increase their expression levels. Conversely, for risk-associated genes, we identified drugs that reduce their expression levels.

### Molecular docking

We obtained the two-dimensional structures of each small molecule ligand drug from the PubChem database (PDB, https://www.rcsb.org/). The structures were imported into Chem3D software to calculate the minimum free energy and convert them into three-dimensional (3D) structures. The 3D structures of the target proteins, the receptors, were obtained from the RCSB Protein Data Bank (PDB, https://www.rcsb.org/). The structures were imported into PyMOL to remove water molecules and ligands. The AutoDock Tool (version 1.5.6) was used to prepare the receptors and ligands by obtaining their PDBQT formats, as well as creating a 3D grid box for the receptor for subsequent molecular docking simulations. Molecular docking analysis was performed using AutoDock Vina (Version 1.1.2). Finally, the best predicted binding site was visualized using PyMOL (https://www.pymol.org/). A binding energy of less than − 5 kcal/mol was defined as indicative of effective ligand-receptor binding, and the binding energy less than − 7 kcal/mol indicated strong binding activity.

## Results

### Mendelian randomisation

The genetic variation range used as IVs for each microbial taxonomic group exposure consists of 3 to 22 SNPs, with F-statistics ranging from 17 to 29, indicating no evidence of weak instrument bias. The MR-PRESSO global test also provided no evidence of pleiotropic effects (*p* > 0.05). (Additional file 1: Table S1).

**Fig. 2:**
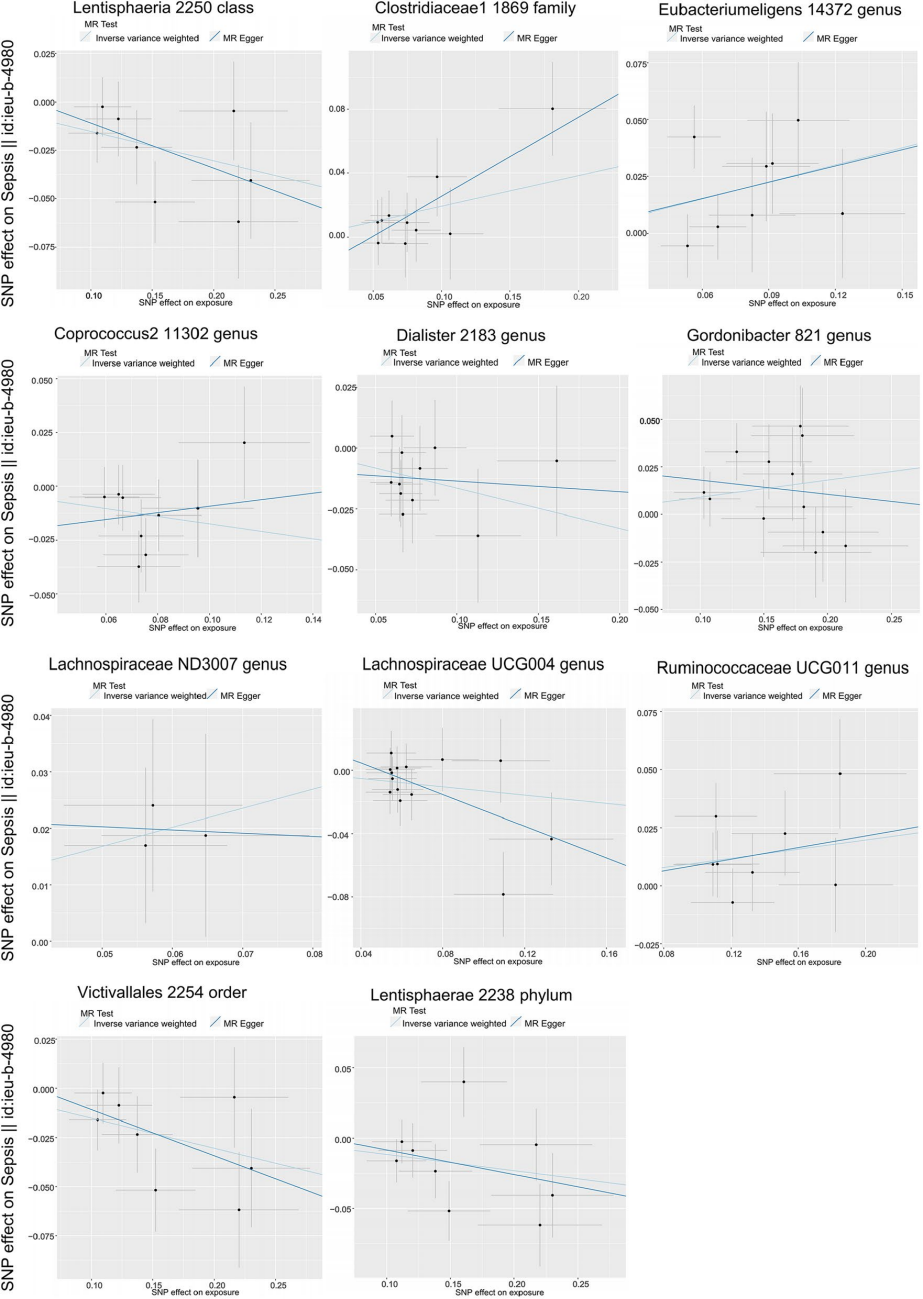
Scatter plots for the causal association between gut microbiota and sepsis

**Fig. 3:**
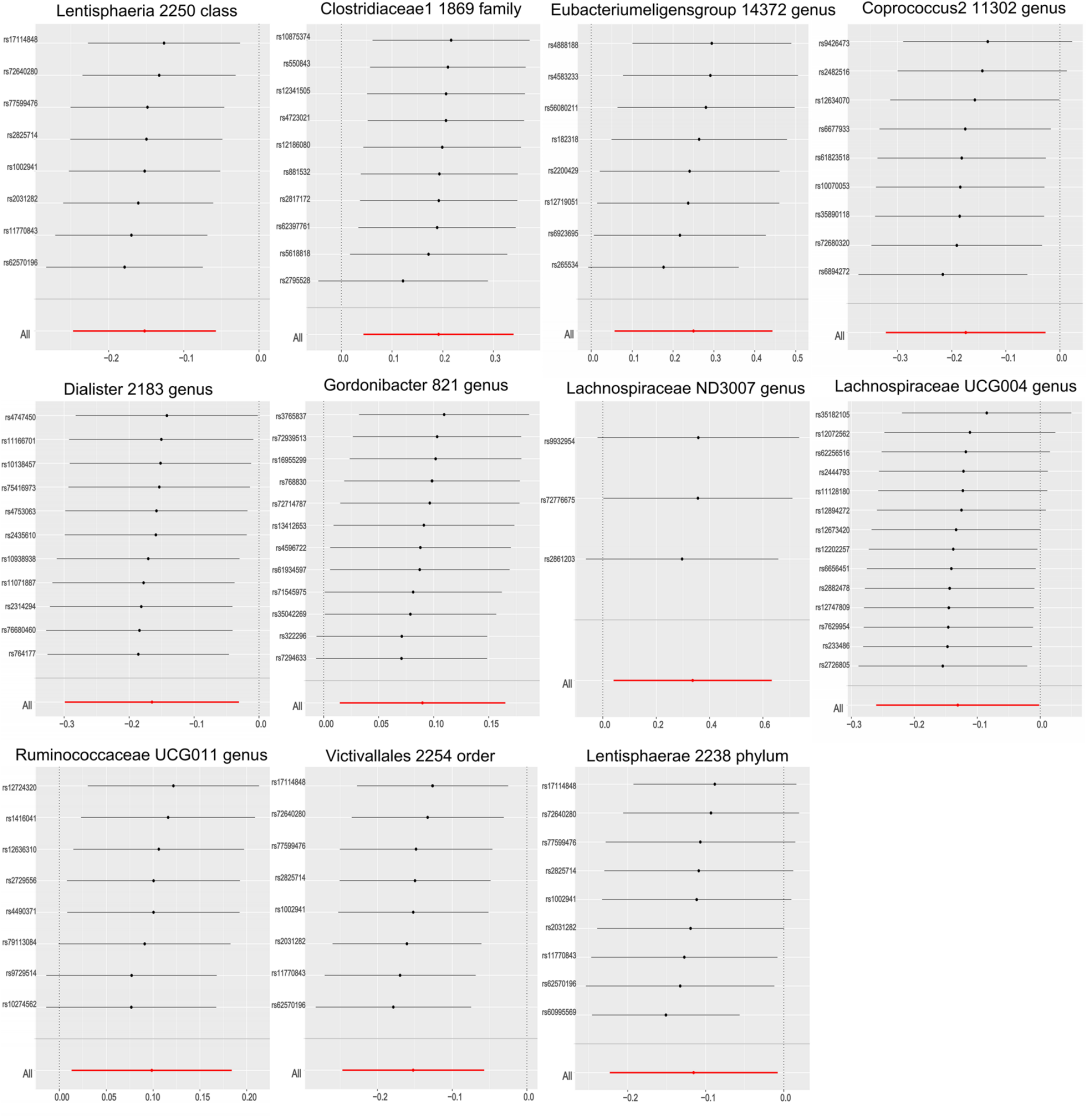
Leave-one-out plots for the causal association between gut microbiota and sepsis

According to the IVW MR analysis, we identified 11 taxonomic groups that had a causal impact on the primary outcome, sepsis. For the 28-day survival outcome in sepsis, 10 taxonomic groups showed a causal effect. In relation to sepsis, we found that increased abundance of Lentisphaeria class (odds ratio [OR], 0.859; 95% confidence interval [CI] 0.781–0.944; *p* = 0.002), Coprococcus2 genus (OR, 0.840; 95% CI 0.725–0.974; *p* = 0.021), Dialiste genus (OR, 0.848; 95% CI 0.742–0.970; *p* = 0.016), Lachnospiraceae UCG004 genus (OR, 0.877; 95% CI 0.771–0.998; *p* = 0.047), Victivallales order (OR, 0.859; 95% CI 0.781–0.944; *p* = 0.002), and Lentisphaerae phylum (OR, 0.891; 95% CI 0.800–0.992; *p* = 0.035) were positively associated, while Clostridiaceae1 family (OR, 1.211; 95% CI 1.045–1.404; *p* = 0.011), Eubacterium eligens genus (OR, 1.284; 95% CI 1.058–1.558; *p* = 0.011), Gordonibacter genus (OR, 1.094; 95% CI 1.015–1.180; *p* = 0.019), Lachnospiraceae ND3007 genus (OR, 1.400; 95% CI 1.040–1.883; *p* = 0.026), and Ruminococcaceae UCG011 genus (OR, 1.103; 95% CI 1.013–1.202; *p* = 0.024) had negative causal effects (Fig. 2, Table 1). There was no evidence of heterogeneity among the genetic IVs for these microbial taxa (Additional file 2: Table S2). For the 28-day survival outcome in sepsis, an increased abundance of Lentisphaeria class (OR, 0.859; 95% CI 0.7510–0.944; *p* = 0.002), Coprococcus1 genus (OR, 0.693; 95% CI 0.499–0.961; *p* = 0.028), Coprococcus2 genus (OR, 0.539; 95% CI 0.310–0.936; *p* = 0.028), Lachnospiraceae FCS020 genus (OR, 0.738; 95% CI 0.550–0.989; *p* = 0.042), Lentisphaerae phylum (OR, 0.721; 95% CI 0.559–0.930; *p* = 0.012), and Victivallales order (OR, 0.678; 95% CI 0.531–0.865; *p* = 0.002) were positively associated, while Bacteroidia class (OR, 1.485; 95% CI 1.069–2.063; *p* = 0.018), Family XIII (OR, 1.563; 95% CI 1.058–2.311; *p* = 0.025), Terrisporobacter genus (OR, 1.434; 95% CI 1.016–2.023; *p* = 0.040), and Bacteroidales order (OR, 1.485; 95% CI 1.069–2.063; *p* = 0.018) had negative causal effects (Additional file 8: Figure S1; Table 2). There was no evidence of heterogeneity among the genetic IVs for these microbial taxa (Additional file 3: Table S3). Nine taxonomic groups showed causal effects on COPD/asthma/ILD related pneumonia or pneumonia-derived septicaemia, with five groups showing a positive causal relationship. Eight taxonomic groups showed causal effects on COPD/asthma-related pneumonia or pneumonia-derived septicaemia, with four groups showing a positive causal relationship. Nine taxonomic groups showed causal effects on Asthma-related pneumonia or sepsis, with three groups showing a positive causal relationship (Additional file 9: Figure S2, Additional file 10: Figure S3, Additional file 11: Figure S4; Additional file 4: Table S4, Additional file 5: Table S5). None of the MR-Egger regression intercepts deviated significantly from zero, indicating no evidence of horizontal pleiotropy (all intercepts p > 0.05) (Additional file 5: Table S5, Additional file 6: Table S6). Additionally, the leave-one-out analysis showed no marked difference in causal estimations of each gut microbiota on sepsis and other outcome, suggesting that none of the identified causal associations were driven by any single IV (Fig. 3, Additional file 12: Figure S5, Additional file 13: Figure S6, Additional file 14: Figure S7, Additional file 15: Figure S8). In the reverse MR analysis, there was no evidence of a causal relationship between these diseases and the gut microbial taxa.

**Table 1:** MR estimates for the association between gut microbiota and sepsis

Bacterial taxa (exposure)	MR method	NSNP	OR	L 95% CI	U 95% CI	*p*-value
Lentisphaeria 2250 class	IVW	8	0.8588	0.7812	0.9442	0.0017
	MR Egger	8	0.7915	0.5705	1.0982	0.2113
	Weighted median	8	0.8538	0.7510	0.7510	0.0158
	Weighted mode	8	0.8858	0.7272	1.0789	0.2672
	Simple mode	8	0.8720	0.7102	1.0706	0.2321
Clostridiaceae1 1869 family	IVW	10	1.2112	1.0447	1.4043	0.0111
	MR Egger	10	1.6419	1.0782	2.5000	0.0496
	Weighted median	10	1.1884	0.9729	1.4516	0.0908
	Weighted mode	10	1.1289	0.7874	1.6186	0.5260
	Simple mode	10	1.1265	0.8128	1.5613	0.4924
Eubacteriumeligens group 14372 genus	IVW	8	1.2841	1.0585	1.5579	0.0112
	MR Egger	8	1.2694	0.5842	2.7582	0.5689
	Weighted median	8	1.1017	0.8646	1.4038	0.4333
	Weighted mode	8	1.0679	0.7756	1.4703	0.6994
	Simple mode	8	1.0820	0.7202	1.6255	0.7155
Coprococcus2 11302 genus	IVW	9	0.8402	0.725	0.9736	0.0206
	MR Egger	9	1.1628	0.4869	2.7768	0.7441
	Weighted median	9	0.9008	0.734	1.1053	0.3168
	Weighted mode	9	0.9140	0.6786	1.2311	0.5705
	Simple mode	9	0.9140	0.6687	1.2493	0.5883
Dialister 2183 genus	IVW	11	0.8480	0.7416	0.9695	0.0158
	MR Egger	11	0.9571	0.5600	1.6358	0.8762
	Weighted median	11	0.8339	0.6991	0.9947	0.0435
	Weighted mode	11	0.7639	0.5760	1.0132	0.0911
	Simple mode	11	0.7609	0.5729	1.0106	0.0884
Gordonibacter 821 genus	IVW	12	1.0941	1.0148	1.1795	0.0191
	MR Egger	12	0.9278	0.6738	1.2775	0.6559
	Weighted median	12	1.0913	0.9839	1.2103	0.0984
	Weighted mode	12	1.0011	0.8096	1.2380	0.9920
	Simple mode	12	1.0073	0.8250	1.2299	0.9442
Lachnospiraceae ND3007 genus	IVW	3	1.3999	1.0405	1.8834	0.0263
	MR Egger	3	0.9446	0.0064	138.9848	0.9857
	Weighted median	3	1.3540	0.9313	1.9686	0.1125
	Weighted mode	3	1.3457	0.8798	2.0584	0.3044
	Simple mode	3	1.3446	0.9019	2.0046	0.3044
Lachnospiraceae UCG004 genus	IVW	14	0.8771	0.7706	0.9983	0.0471
	MR Egger	14	0.6067	0.3628	1.0145	0.081
	Weighted median	14	0.9568	0.8049	1.1375	0.617
	Weighted mode	14	1.0086	0.7567	1.3443	0.9546
	Simple mode	14	1.0086	0.7524	1.3518	0.9554
Ruminococcaceae UCG011 genus	IVW	8	1.1036	1.1035	1.2020	0.0237
	MR Egger	8	1.1335	0.7243	1.7737	0.6032
	Weighted median	8	1.0877	0.9657	1.2252	0.1658
	Weighted mode	8	1.0582	0.8934	1.2534	0.5333
	Simple mode	8	1.0656	0.8809	1.2891	0.5337
Victivallales 2254 order	IVW	8	0.8588	0.7812	0.9442	0.0017
	MR Egger	8	0.7915	0.5705	1.098	0.2113
	Weighted median	8	0.8538	0.7506	0.9712	0.0162
	Weighted mode	8	0.8858	0.7225	1.0859	0.2814
	Simple mode	8	0.8719	0.7125	1.0672	0.2255
Lentisphaerae 2238 phylum	IVW	9	0.8910	0.8001	0.9922	0.0354
	MR Egger	9	0.8396	0.5525	1.2759	0.4399
	Weighted median	9	0.8729	0.7722	0.9867	0.0296
	Weighted mode	9	0.8921	0.7332	1.0855	0.2871
	Simple mode	9	0.8796	0.7219	1.0718	0.2390

*IVW* inverse variance weighted, *MR* Mendelian randomisation, *NSNP* number of single-nucleotide polymorphisms, *OR* odds ratio, *L 95% CI* lower 95% confidence interval, *U 95% CI* upper 95% confidence interval

**Table 2:** MR estimates for the association between gut microbiota and survival of sepsis

Bacterial taxa (exposure)	MR method	NSNP	OR	L 95% CI	U 95% CI	*p*-value
Bacteroidia 912 class	IVW	14	1.4850	1.0691	2.0625	0.0183
	MR Egger	14	1.2214	0.6043	2.4686	0.5877
	Weighted median	14	1.4842	0.9351	2.356	0.0939
	Weighted mode	14	1.4448	0.7893	2.6447	0.2543
	Simple mode	14	1.1944	0.5386	2.6487	0.6691
Lentisphaeria 2250 class	IVW	8	0.6779	0.5311	0.8652	0.0018
	MR Egger	8	0.7098	0.2859	1.7621	0.4879
	Weighted median	8	0.7265	0.5314	0.993	0.0451
	Weighted mode	8	0.8569	0.5109	1.4374	0.5769
	Simple mode	8	0.8344	0.5005	1.3912	0.5101
FamilyXIII 1957 family	IVW	10	1.5632	1.0575	2.3105	0.0250
	MR Egger	10	5.6288	1.4367	22.0535	0.0381
	Weighted median	10	1.3089	0.7726	2.2175	0.3169
	Weighted mode	10	1.0497	0.4102	2.6862	0.9217
	Simple mode	10	1.0497	0.4431	2.4868	0.9147
Coprococcus1 11301 genus	IVW	12	0.6926	0.4992	0.9610	0.0279
	MR Egger	12	0.5276	0.2429	1.1458	0.1371
	Weighted median	12	0.8395	0.5241	1.3446	0.4666
	Weighted mode	12	0.9983	0.4317	2.3085	0.9968
	Simple mode	12	1.0094	0.4363	2.3355	0.9829
Coprococcus2 11302 genus	IVW	9	0.5390	0.3105	0.9358	0.0281
	MR Egger	9	2.0173	0.0717	56.7457	0.6925
	Weighted median	9	0.5847	0.3329	1.0271	0.0619
	Weighted mode	9	0.4732	0.1710	1.3097	0.1879
	Simple mode	9	0.4580	0.1524	1.3765	0.2017
Lachnospiraceae FCS020 genus	IVW	13	0.7375	0.5499	0.9891	0.0420
	MR Egger	13	0.4264	0.2033	0.8946	0.0455
	Weighted median	13	0.7537	0.5004	1.1351	0.1759
	Weighted mode	13	0.8318	0.4409	1.5692	0.5800
	Simple mode	13	0.9349	0.4437	1.9698	0.8625
Terrisporobacter 11348 genus	IVW	5	1.4337	1.0160	2.0231	0.0403
	MR Egger	5	1.0294	0.4008	2.6442	0.9556
	Weighted median	5	1.3120	0.8415	2.0454	0.2308
	Weighted mode	5	1.3096	0.7862	2.1815	0.3588
	Simple mode	5	1.3068	0.7591	2.2496	0.3889
Bacteroidales 913 order	IVW	14	1.4850	1.0691	2.0625	0.0183
	MR Egger	14	1.2214	0.6043	2.4686	0.5877
	Weighted median	14	1.4842	0.9295	2.3700	0.0981
	Weighted mode	14	1.4448	0.8122	2.5699	0.2326
	Simple mode	14	1.1944	0.5704	2.5013	0.6453
Victivallales 2254 order	IVW	8	0.6779	0.5311	0.8652	0.0018
	MR Egger	8	0.7098	0.2859	1.7621	0.4879
	Weighted median	8	0.7264	0.5321	0.9919	0.0443
	Weighted mode	8	0.8569	0.5266	1.3946	0.5540
	Simple mode	8	0.8344	0.4922	1.4147	0.5231
Lentisphaerae 2238 phylum	IVW	9	0.7209	0.5588	0.9300	0.0118
	MR Egger	9	0.7852	0.2905	2.1224	0.6481
	Weighted median	9	0.7947	0.5853	1.079	0.1409
	Weighted mode	9	0.8711	0.5304	1.4305	0.6003
	Simple mode	9	0.8577	0.5125	1.4353	0.5750

*IVW* inverse variance weighted, *MR* Mendelian randomisation, *NSNP* number of single-nucleotide polymorphisms, *OR* odds ratio, *L 95% CI* lower 95% confidence interval, *U 95% CI* upper 95% confidence interval

### Genes and functions

**Fig. 4:**
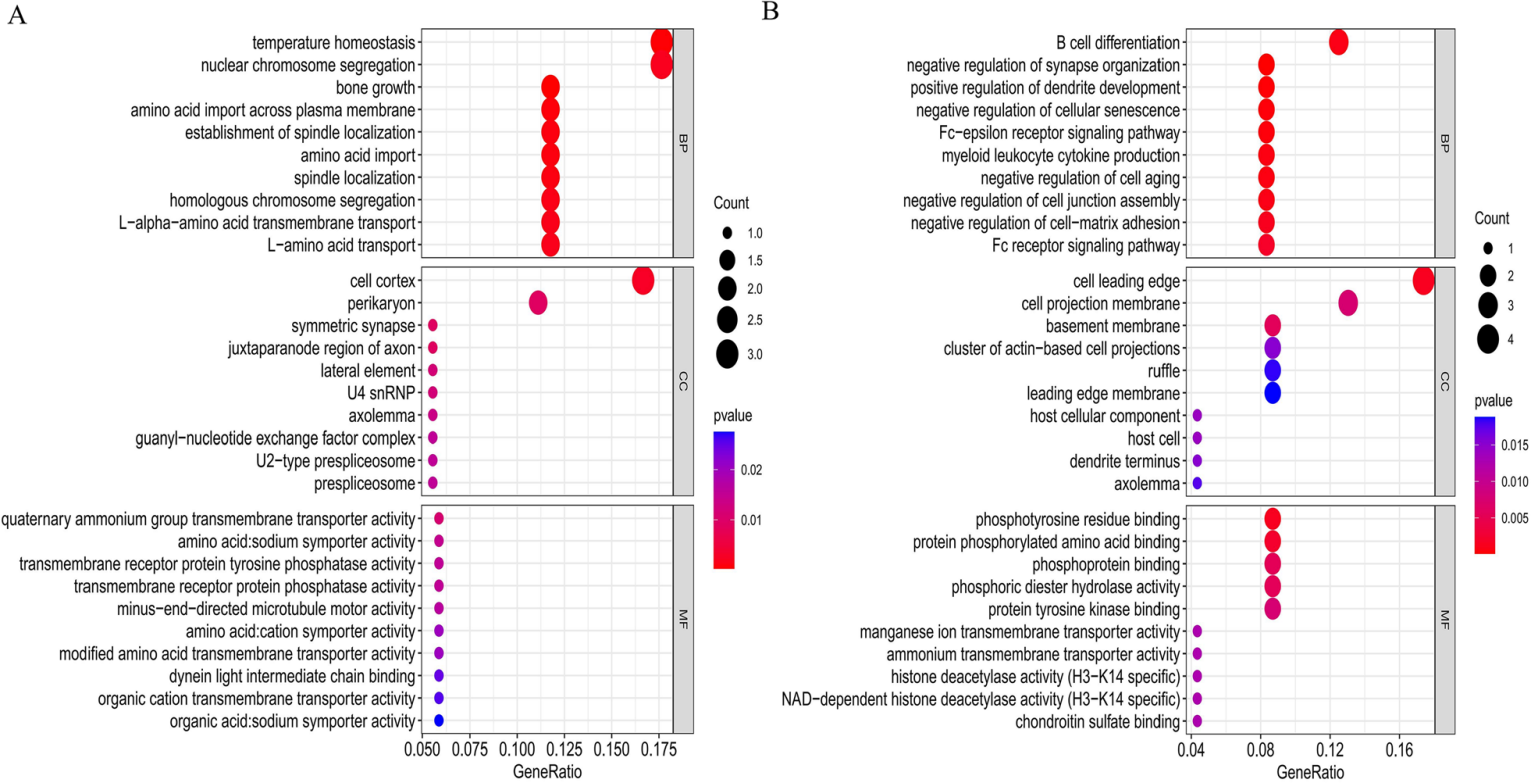
Enrichment analysis of gene ontology (GO) terms for potential disease-related key genes associated with gut microbiota

The correspondence between SNPs and genes and their functions is presented in Additional file 6: Table S6. GO analysis revealed that gut microbiota associated with favorable outcomes in diseases may involve activities such as bile acid/organic hydroxy compound/monocarboxylic acid/lipid/carboxylic acid/organic acid transmembrane transporter activity, protein N-terminus binding, lipid transporter activity, monocarboxylic acid transport, regulation of glutamate secretion, glutamate secretion, and regulation of respiratory gaseous exchange. On the other hand, gut microbiota associated with unfavorable outcomes in diseases may involve activities such as lipase/lysophospholipase activity, actin/phospholipase/phosphotyrosine residue/protein phosphorylated amino acid binding, regulation of inflammatory response, axo-dendritic transport, negative regulation of long-term synaptic potentiation, monoacylglycerol metabolic process, positive regulation of histone deacetylation, retrograde axonal transport, and Fc-epsilon receptor signaling pathway (Fig. 4). No significant enrichment pathway was found in KEGG pathway enrichment analysis.

### Single-cell analysis results

**Fig. 5:**
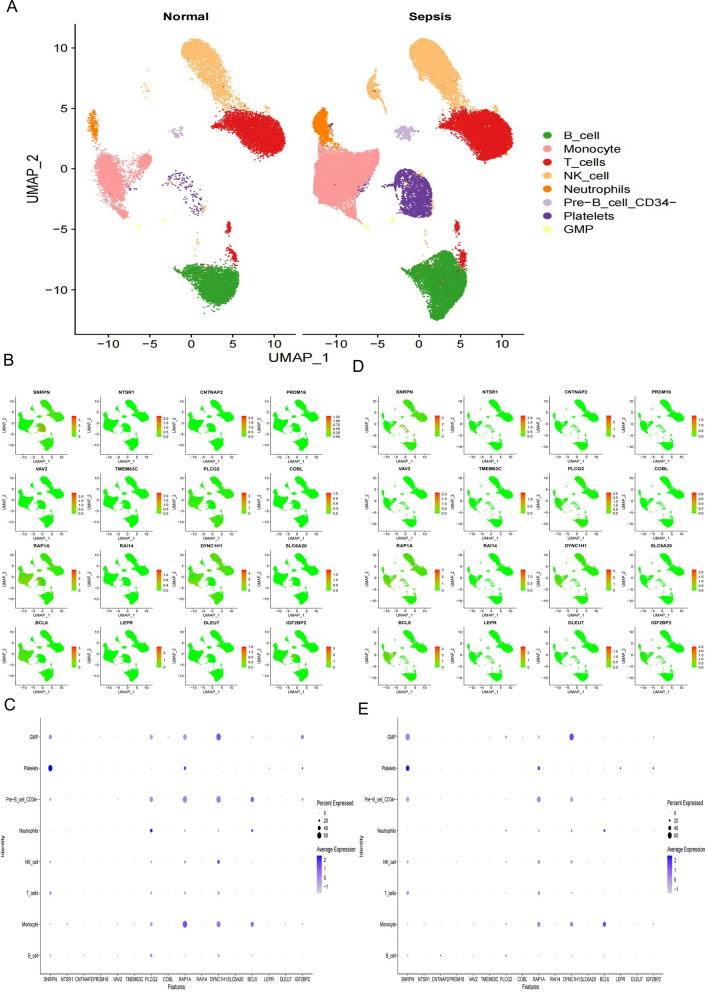
Expression of potential key genes of sepsis associated with gut microbiota in sepsis single-cell data. **A** Uniform manifold approximation and projection (UMAP) clustering map of human peripheral blood mononuclear cells from 2 healthy controls, 4 survivors, and 6 non-survivors of gram-negative sepsis patients. **B**, **C** Expression of potential sepsis key genes associated with gut microbiota in each cell type of sepsis samples. **D**, **E** Expression of potential sepsis key genes associated with gut microbiota in each cell type of health samples

**Fig. 6:**
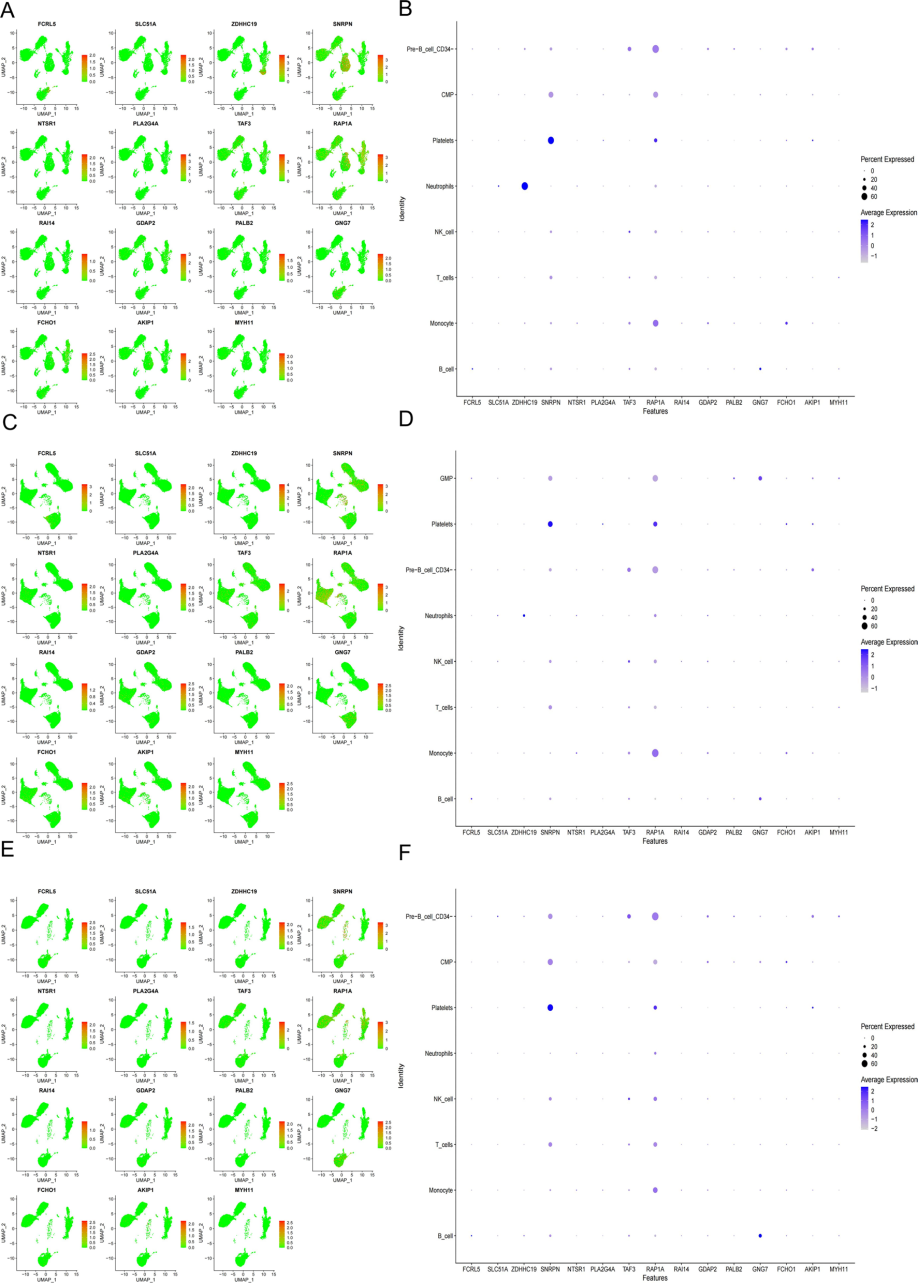
Expression of potential key genes of non-survivors of sepsis associated with gut microbiota in sepsis single-cell data. **A**, **B** Expression of potential key genes in each cell type of non-survivors of gram-negative sepsis samples. **C**, **D** Expression of potential key genes in each cell type of survivors of sepsis samples. **E**, **F** Expression of potential key genes in each cell type of health samples

We analyzed scRNA-seq data from 12 samples, including human peripheral blood mononuclear cells from 2 healthy controls, 6 survivors, and 4 non-survivors of gram-negative sepsis patients. After preprocessing the data with strict quality control metrics, we visualized the high-dimensional scRNA-seq data using UMAP technique based on the top 15 principal components. Subsequently, we successfully classified the cells into 14 subclusters and annotated them into recognizable cell types using the SingleR R package. The major cell types included B cells, Monocytes, T cells, NK cells, Platelets, Neutrophils, GMP granulocyte-monocyte progenitors, Pre-B cells (CD34−), and BM Bone marrow (Fig. 5A). We then analyzed the genes associated with genetic variations in 11 intestinal flora groups, which served as instrumental variables (IVs) for sepsis. We examined the expression of these genes in the cells. For sepsis, we found that PLCG2 was upregulated in immune cells during sepsis compared to healthy individuals, BCL6 was upregulated in Pre-B cells (CD34−) during sepsis, and IGF2BP2 was upregulated in GMP cells (Fig. 5B–E). Regarding sepsis mortality at 28 days, we observed that ZDHHC19 had the lowest expression in Platelets of healthy individuals, increased expression in Platelets of sepsis survivors at 28 days, and the highest expression in Platelets of sepsis non-survivors at 28 days. Additionally, compared to normal samples, SNRPN exhibited reduced expression in Pre-B cells (CD34−) and T cells of sepsis (Fig. 6). For other infectious diseases, we found that FMN1 had lower expression in Pre-B cells (CD34−) and Monocytes of healthy individuals compared to sepsis patients, MGLL had lower expression in Platelets of healthy individuals compared to sepsis patients, and APP and CE NPN had lower expression in Pre-B cells (CD34−) of healthy individuals compared to sepsis patients (Additional file 16: Figure S9, Additional file 17: Figure S10, Additional file 18: Figure S11).

### Bulk RNA analysis results

Using the GSE65682 dataset, we performed differential gene expression analysis between healthy individuals and sepsis patients using limma. We identified significant upregulation of NTSR1, BCL6, ZDHHC19, MGLL, and ALPK1 in sepsis compared to healthy individuals, while VAV2 and SATB1 were significantly downregulated in sepsis (Additional file 19: Figure S12A–G). Furthermore, compared to sepsis patients who survived at 28 days, we observed a significant downregulation of FCHO1 in sepsis patients who died at 28 days (Additional file 19: Figure S12H). These findings are consistent with Mendelian randomization and single-cell analysis results.

### Molecular docking

**Fig. 7:**
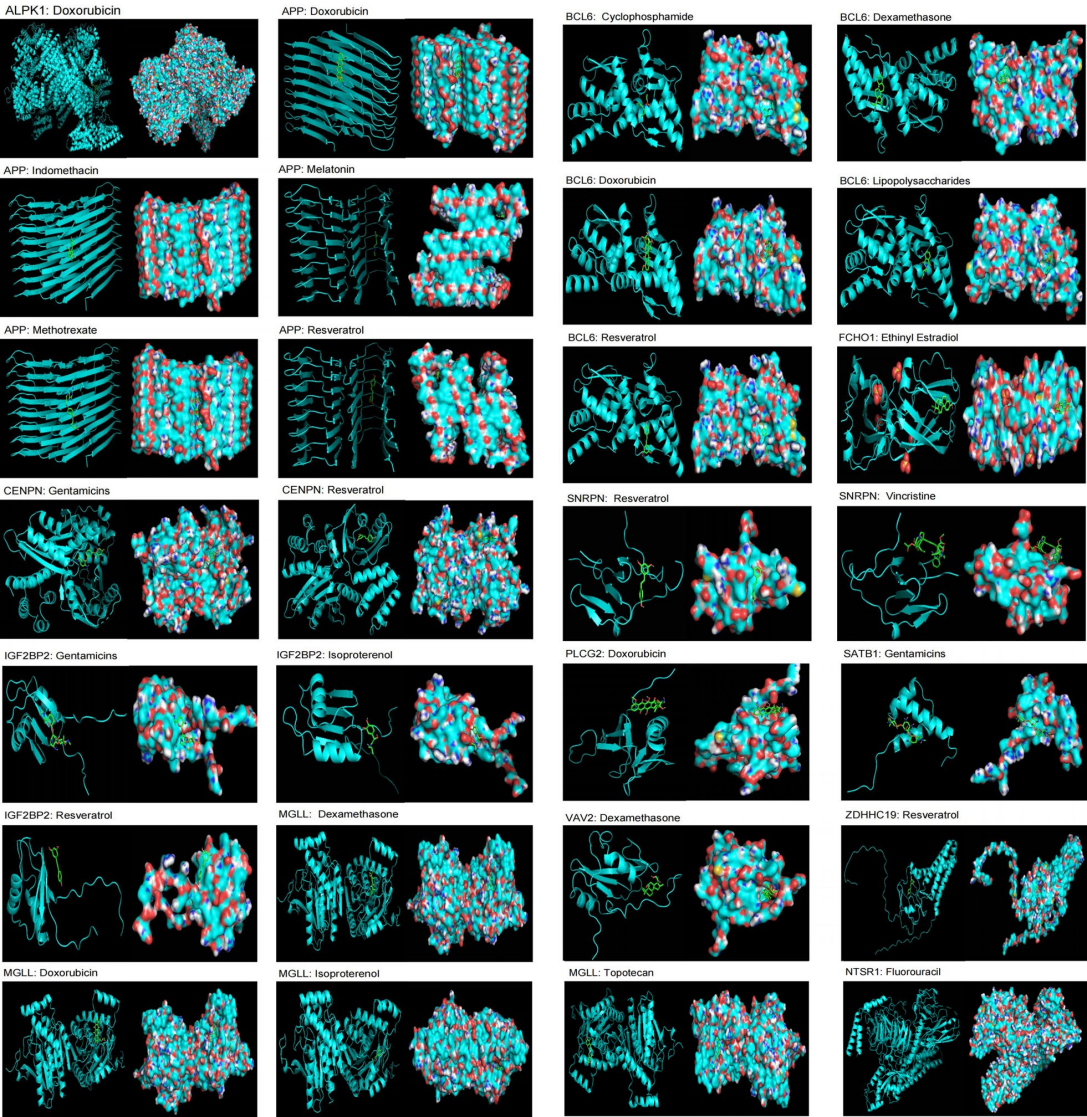
Molecular docking diagram of potential therapeutic targets for the disease and potential therapeutic drugs

In sepsis, we found that Fluorouracil can decrease the expression of NTSR1. Cyclophosphamide, Dexamethasone, Doxorubicin, Lipopolysaccharides, and Resveratrol can reduce the expression of BCL6. Resveratrol can decrease the expression of ZDHHC19. Cisplatin, Dexamethasone, Doxorubicin, Isoproterenol, and Topotecan can decrease the expression of MGLL. Doxorubicin can decrease the expression of ALPK1 and PLCG2. Doxorubicin, Indomethacin, Melatonin, Methotrexate, and Resveratrol can decrease the expression of APP. Resveratrol and Vincristine can reduce the expression of SNRPN. Dexamethasone can increase the expression of VAV2. Gentamicins can increase the expression of SATB1. Ethinyl Estradiol can increase the expression of FCHO1. Gentamicins, Isoproterenol, Methotrexate, and Resveratrol can increase the expression of IGF2BP2. Gentamicins and Resveratrol can increase the expression of CENPN (Fig. 7). The binding affinities between the small molecule ligands and their targets are shown in the Additional file 7: Table S7.

## Discussion

In this study, we performed a two-sample MR analysis using summary statistics data from the largest GWAS meta-analysis of gut microbiota and sepsis, conducted by the MiBioGen Consortium. The aim was to evaluate the causal relationship between gut microbiota and sepsis, as well as its related diseases. It is worth mentioning that choosing bidirectional MR in our study aims for a comprehensive understanding of the intricate relationship between gut microbiota and sepsis. This approach allows us to assess how the microbiota influences sepsis and vice versa within a unified framework, revealing potential biological mechanisms. Additionally, bidirectional MR helps consider reverse causation systematically, crucial for establishing causality while identifying mechanisms of sepsis-induced microbiota changes. Emphasizing these advantages in the discussion highlights the innovation and depth of our study, ensuring reliable causal inferences by effectively controlling potential confounding factors through consideration of common genetic factors.

We identified several gut microbial taxa that showed suggestive protective effects against sepsis, including Lentisphaeria class, Coprococcus2 genus, Dialiste genus, Lachnospiraceae UCG004 genus, Victivallales order, and Lentisphaerae phylum. On the other hand, some taxa showed suggestive harmful effects, including Clostridiaceae1 family, Eubacterium eligens genus, Gordonibacter genus, Lachnospiraceae ND3007 genus, and Ruminococcaceae UCG011 genus. For the 28-day survival outcome in sepsis, Lentisphaeria class, Coprococcus1 genus, Coprococcus2 genus, Lachnospiraceae FCS020 genus, Lentisphaerae phylum, and Victivallales order may have protective effects, while Bacteroidia class, Family XIII, Terrisporobacter genus, and Bacteroidales order may have harmful effects.

Coprococcus, an important member of the Firmicutes phylum and Lachnospiraceae family, actively ferments carbohydrates and is one of the key producers of butyric acid, similar to *Faecalibacterium prausnitzii*, a beneficial microbial species associated with intestinal health and immune system balance [49]. Coprococcus can serve as a microbial biomarker for assessing gastrointestinal health in humans [50–52]. The genus Coprococcus may contribute to immune response suppression and reduce the severity of allergic reactions. In our results, Coprococcus2 genus showed favorable associations with both sepsis and sepsis survival outcomes. In sepsis, the inflammatory response triggered by pathogenic infection may exceed the necessary defense against infection and result in damage to normal tissues and organs. This excessive immune response typically involves multiple immune cells and molecules, including leukocytes, cytokines, and chemokines. This may be a mechanism through which Coprococcus genus exerts its beneficial effects. Lachnospiraceae is a family of obligate anaerobic bacteria within the phylum Firmicutes that readily forms spores. They ferment various plant polysaccharides into short-chain fatty acids (such as butyrate and acetate) and alcohols (such as ethanol) [53, 54]. Some studies suggest that Lachnospiraceae bacteria may play a role in regulating intestinal inflammation [55, 56]. Certain members of the Lachnospiraceae family can modulate the intestinal environment by producing short-chain fatty acids, such as butyrate, to maintain intestinal barrier function and inhibit inflammatory responses [57]. Butyrate can serve as an energy source for intestinal epithelial cells and affect the function of immune cells, thereby reducing the intensity of inflammation [58, 59]. Additionally, the abundance and diversity of Lachnospiraceae bacteria have been associated with the occurrence and development of inflammatory bowel diseases, indicating that an imbalance in Lachnospiraceae bacteria may be related to exacerbated intestinal inflammation [60]. Our research findings are consistent with previous studies, suggesting a favorable causal relationship between Lachnospiraceae UCG004 genus and Lachnospiraceae FCS020 genus and sepsis and its survival outcomes, while Lachnospiraceae ND3007 genus showed unfavorable effects. Eubacterium genus bacteria have been shown to play critical roles in various aspects, including bile acid and cholesterol transformation, involvement in oxalate metabolism, promotion of anti-inflammatory molecule production, prevention of allergic airway inflammation, modulation of insulin secretion, and regulation of lipid metabolism [61, 62]. Ruminococcus is a common genus of gut bacteria that plays a significant role in the digestion and metabolism of resistant starch. However, Ruminococcus is also associated with various gastrointestinal diseases, immune-related disorders, and neurological disorders [63]. Some studies have found that the quantity and activity of Ruminococcus in the intestines of patients with these diseases may vary, thereby affecting intestinal health and the degree of inflammation [64]. Abnormal states of Ruminococcus may be associated with the occurrence and development of immune-related diseases, such as allergies, eczema, and asthma [65]. The specific mechanisms are not yet clear, but studies suggest that Ruminococcus may be involved in the pathogenesis of immune-mediated diseases by influencing the regulation of the intestinal immune system and inflammatory responses. Furthermore, dysbiosis of Ruminococcus has also been linked to neurological disorders such as autism and depression [66, 67]. The gut-brain axis may play an important role in the mechanisms underlying these diseases, and the abnormal state of Ruminococcus may impact neural function through its effects on intestinal-brain signaling and inflammatory responses. Bacteroides is a common genus of bacteria in the human gut and has a symbiotic relationship with humans, playing a crucial role in maintaining gut health and function [68]. They assist in the breakdown of complex carbohydrates in food and produce essential nutrients and energy for the body. In certain cases, Bacteroides may be one of the pathogens leading to sepsis [69]. Intestinal perforation or rupture, dysbiosis of the gut microbiota, and compromised immune function can allow gut bacteria like Bacteroides to enter the abdominal cavity or bloodstream, triggering infection and sepsis. However, the relationship between most bacterial populations and diseases has not been fully investigated. The complexity of the gut microbiota and inter-individual variations make research challenging. Therefore, further studies are needed to understand the role of gut bacteria in infection-related diseases like sepsis, in order to develop potential therapeutic strategies.

Furthermore, we integrated GWAS and eQTL data into MR analysis to explore genes that may have potential causal relationships with sepsis. To better understand the biological functions of these genes in the disease, we conducted GO and KEGG analyses. We found that protective gut microbiota may exert their effects through the Glutamate and lipid metabolic pathways, while harmful gut microbiota may exert their effects through lysophospholipase activity, regulation of inflammatory response, axo-dendritic transport, negative regulation of long-term synaptic potentiation, monoacylglycerol metabolic process, positive regulation of histone deacetylation, retrograde axonal transport, and Fc-epsilon receptor signaling pathway. Single-cell analysis revealed increased expression of PLCG2 in immune cells of sepsis compared to healthy individuals, increased expression of BCL6 in sepsis Pre-B cell CD34−, increased expression of IGF2BP2 in GMP, increased expression of FMN1 in sepsis Pre-B cell CD34− and Monocytes, increased expression of MGLL in sepsis Platelets, and increased expression of APP and CENPN in sepsis Pre-B cell CD34−. SNRPN showed decreased expression in sepsis Pre-B cell CD34− and T cells. ZDHHC19 showed the lowest expression in Platelets of healthy individuals, increased expression in Platelets of sepsis survivors at 28 days, and the highest expression in Platelets of sepsis non-survivors at 28 days. Additionally, bulk RNA analysis revealed significant increases in NTSR1, BCL6, ZDHHC19, MGLL, and ALPK1 in sepsis, and significant decreases in VAV2 and SATB1 in sepsis; FCHO1 showed a significant decrease in sepsis patients who did not survive compared to those who survived at 28 days. These results suggest that PLCG2, BCL6, IGF2BP2, FMN1, MGLL, APP, CENPN, SNRPN, ZDHHC19, NTSR1, ALPK1, VAV2, SATB1, and FCHO1 may represent novel genes involved in the potential pathogenesis of sepsis and potential therapeutic targets. Therefore, we analyzed potential therapeutic drugs and performed molecular docking with these genes and their targeted drugs. Dexamethasone, Doxorubicin, Gentamicins, and Resveratrol all showed three or more action targets, indicating their potential for disease treatment. Among the identified drugs, Dexamethasone, Gentamicins, and Resveratrol stand out due to their distinct roles and significant implications in the context of sepsis. The observed significant upregulation of NTSR1, BCL6, ZDHHC19, MGLL, and ALPK1 in sepsis compared to healthy individuals, along with the notable downregulation of VAV2 and SATB1 in sepsis, provides a comprehensive picture of the altered gene expression landscape during sepsis. Particularly, the significant downregulation of FCHO1 in sepsis patients who died at 28 days underscores its potential relevance to adverse clinical outcomes. We will focus on three key ligands with plausible biological roles: Dexamethasone, Gentamicins, and Resveratrol. Dexamethasone, a potent corticosteroid, is known for its anti-inflammatory and immunomodulatory effects. Gentamicins, as a class of aminoglycoside antibiotics, exhibit broad-spectrum antibacterial activity. Resveratrol, a natural polyphenol, has been studied for its antioxidant and anti-inflammatory properties. Ethinyl estradiol, identified through molecular docking, has been shown to increase the expression of FCHO1. While this observation is intriguing, we recognize the need for further exploration of the role of ethinyl estradiol in the context of sepsis and its potential therapeutic implications.

Phospholipase C gamma 2 (PLCG2) is involved in intracellular signaling pathways and has been associated with immune responses and inflammation [70, 71]. It plays a role in regulating inflammatory signaling cascades and immune cell activation [72]. Aberrant PLCG2 activity or mutations in PLCG2 gene have been linked to certain inflammatory disorders, such as autoinflammatory diseases and autoimmune conditions [73]. B-cell lymphoma 6 (BCL6) is a transcriptional repressor and is primarily known for its role in B cell development and function. While BCL6 is not directly implicated in inflammation and sepsis, it plays a role in modulating immune responses and can influence the balance between pro-inflammatory and anti-inflammatory signaling pathways [74, 75]. Insulin-like growth factor 2 mRNA-binding protein 2 (IGF2BP2) is an RNA-binding protein that regulates the stability and translation of specific mRNAs [76]. Although IGF2BP2 has been primarily studied in the context of diabetes and cancer, its association with inflammation and sepsis is not well-established [77, 78]. Formin 1 (FMN1) is a member of the formin family of proteins that are involved in actin cytoskeleton organization [79]. While FMN1’s role in inflammation and sepsis has not been extensively studied, it has been implicated in cell migration and immune cell function, suggesting a potential involvement in inflammatory processes. Monoglyceride lipase (MGLL) is an enzyme involved in the breakdown of endocannabinoids and lipid metabolism. While MGLL has been primarily studied in the context of metabolism and neurological disorders, its relationship with inflammation and sepsis is not well-characterized [79, 80]. Amyloid precursor protein (APP) is primarily associated with Alzheimer’s disease and the accumulation of amyloid plaques in the brain [81]. Although there is some evidence suggesting a potential link between APP and inflammation, particularly in neuroinflammatory processes, its role in systemic inflammation and sepsis is not well-established [82]. Centromere protein N (CENPN) is a centromere-associated protein involved in chromosome segregation during cell division [83]. Small nuclear ribonucleoprotein polypeptide N (SNRPN) is involved in the processing and function of small nuclear RNAs [84]. While SNRPN’s role in inflammation and sepsis is not well-characterized, alterations in SNRPN gene expression have been observed in certain autoimmune disorders. Zinc finger DHHC-type containing 19 (ZDHHC19) is a member of the DHHC family of palmitoyltransferases, which regulate protein palmitoylation [85]. While ZDHHC19’s specific role in inflammation and sepsis is not well-studied, protein palmitoylation has been implicated in immune cell function and inflammatory signaling pathways [86]. In our study, ZDHHC19 exhibited a distinct expression pattern in platelets, showing the lowest levels in healthy individuals, increased expression in platelets of sepsis survivors at 28 days, and the highest expression in platelets of sepsis non-survivors at 28 days. This dynamic profile prompts questions about ZDHHC19's role in platelet function during sepsis. We hypothesize that elevated ZDHHC19 in septic platelets may correlate with inflammatory severity and clinical outcomes. Sepsis induces an immunosuppressive milieu, and previous studies indicate ZDHHC19's role in Smad3 palmitoylation, activating the TGF-β pathway [87]. In sepsis, increased ZDHHC19 in platelets might modulate TGF-β signaling, impacting immune responses. Considering sepsis severity gradients, exploring the link between ZDHHC19 in platelets and disease gravity becomes crucial. Higher expression in platelets of sepsis non-survivors at 28 days suggests a potential association with adverse clinical outcomes. Drawing from previous studies, we speculate ZDHHC19 overexpression may link to immune-paralysis and immunosenescence, increasing secondary infection risk and mortality [88]. Seeking insights from diverse clinical contexts emphasizes the need to unravel ZDHHC19's mechanisms in platelet function and its potential impact on sepsis progression. Further exploration of the intricate interplay between ZDHHC19, platelets, and the immune response could unveil novel therapeutic targets for attenuating sepsis severity and improving patient outcomes. Neurotensin receptor 1 (NTSR1), primarily associated with neuronal signaling, is also found in immune cells, modulating immune responses [89]. Alpha-protein kinase 1 (ALPK1), a protein kinase regulating cellular processes, includes signal transduction and cell cycle regulation [90]. Vav guanine nucleotide exchange factor 2 (VAV2), a guanine nucleotide exchange factor, impacts cytoskeletal rearrangements and immune cell activation, especially in T cells and macrophages, influencing immune cell functions and cytokine production [91–94]. Special AT-rich binding protein 1 (SATB1), a DNA-binding protein, crucial in immune cell development, particularly T cells, is implicated in T cell differentiation, cytokine production, and immune responses [95–97]. While SATB1’s specific link to systemic inflammation and sepsis is limited, its role in modulating immune responses affects pro-inflammatory and anti-inflammatory pathways. FCH domain only 1 (FCHO1), involved in clathrin-mediated endocytosis, crucial for receptor internalization, has potential relevance in immune cell functions and signaling processes [98, 99]. Further research is needed to understand these genes’ mechanisms in sepsis and their therapeutic potential. The identified drugs through molecular docking for sepsis treatment require additional in vitro, in vivo, and clinical validation.

Of note, the collective relevance and clinical significance of genetic interactions are more important. We should focus on the overarching genetic interactions and their potential clinical significance. Our study uncovered a complex network of genetic associations related to sepsis, implicating multiple genes in the pathogenesis of the disease. In addition, we recognize the relevance of linking our genetic findings to clinical protocols that have proven effective in improving sepsis-related disease outcomes. Specifically, we found the documented benefits of corticosteroids such as hydrocortisone, dexamethasone, or methylprednisolone in managing acute respiratory distress syndrome (ARDS) caused by pneumonia [100–102]. Additionally, aminoglycoside antibiotics have been used in clinical, such as gentamycin or amikacin, in the context of sepsis bacteremia [103]. Furthermore, the emerging evidence regarding the cardiovascular protective role of resveratrol was provided in recent research [104].

It is essential to recognize that despite the significant findings in our study, there are several major limitations that warrant attention: (1) Limitations of Mendelian Randomization Study: Despite employing a Mendelian randomization study design to mitigate the impact of confounding factors, it’s crucial to acknowledge the possibility of other unaccounted potential factors that could influence the results.; Mendelian randomization studies, while powerful, can only reveal correlations and cannot establish causality definitively. As a result, additional functional research is necessary to validate the causal relationship between gut microbiota and sepsis. (2) Limitations in Data Interpretation and Analysis: Technical limitations and interpretational challenges are associated with single-cell transcriptomic sequencing and bulk RNA sequencing data. Challenges include dealing with data noise, identifying and annotating cell types, normalizing data, and employing appropriate statistical methods for differential analysis; Further validation and confirmation are imperative for the accurate interpretation of gene expression in single-cell analysis. (3) Predictive Limitations of Potential Therapeutic Targets: While we have identified certain genes as potential therapeutic targets and conducted molecular docking studies, these findings are preliminary predictions requiring additional research and validation; Molecular docking provides information on potential ligand-target interactions, but its feasibility and efficacy need validation through additional in vitro and in vivo experiments. (4) Sample Selection and Representativeness: The study’s reliance on blood samples from sepsis patients may introduce sample selection bias and limitations. The exclusive focus on blood samples might not fully capture the comprehensive spectrum of sepsis; the sample size may be limited, potentially restricting the study’s ability to comprehensively analyze different sepsis subtypes and severity levels. While our study has provided valuable insights, it is crucial to interpret the findings within the context of these limitations. Future research should address these constraints to enhance the robustness and generalizability of the study’s conclusions.

In summary, our study employed a Mendelian randomization study design to investigate the relationship between gut microbiota and sepsis for the first time. Through this design, we successfully identified gut microbiota associated with sepsis, including both beneficial and harmful microbial communities. Additionally, we utilized eQTL analysis to identify genes associated with sepsis from these instrumental variables (SNPs). This suggests that gut microbiota may exert an impact on the pathogenesis of sepsis through the regulation of these genes. Furthermore, we analyzed and annotated gene expression in different cell types using single-cell transcriptomic sequencing technology in blood samples from sepsis patients. This approach enabled us to observe and compare the expression patterns of these genes in different cells, providing insights into their functions and regulatory mechanisms in the development of sepsis. Moreover, we compared the expression profiles of these genes among healthy individuals, surviving sepsis patients, and deceased sepsis patients using bulk RNA sequencing data. Such comparisons helped us understand the expression changes of these genes in different pathological states and may aid in identifying biomarkers associated with sepsis prognosis and disease severity. Finally, through predictive and molecular docking methods, we explored some genes as potential therapeutic targets and predicted potential therapeutic drugs associated with these targets. This provides insights for the development of personalized treatment strategies for sepsis and offers preliminary candidate targets and drugs for future drug development.

### Supplementary Information

**Additional file 1: Table S1.** MR-PRESSO analysis for the association between gut microbiota and outcomes.

**Additional file 2: Table S2.** The heterogeneity of gut microbiota instrumental variables.

**Additional file 3: Table S3.** The heterogeneity of gut microbiota instrumental variables (28-day survival outcomes for sepsis).

**Additional file 4: Table S4.** MR estimates for the association between gut microbiota and other sepsis related infections.

**Additional file 5: Table S5.** The heterogeneity of gut microbiota instrumental variables (other sepsis related infections).

**Additional file 6: Table S6.** Directional horizontal pleiotropy assessed by intercept term in MR Egger regression of the association between gut microbiota and outcomes.

**Additional file 7: Table S7**. The correspondence between SNP and gene.

**Additional file 8: Figure S1.** Scatter plots for the causal association between gut microbiota and 28-day survival outcomes for sepsis.

**Additional file 9: Figure S2.** Scatter plots for the causal association between gut microbiota and COPD/ asthma/ ILD-related pneumonia or pneumonia-derived septicaemia.

**Additional file 10: Figure S3.** Scatter plots for the causal association between gut microbiota and COPD/ asthma-related pneumonia or pneumonia-derived septicaemia.

**Additional file 11: Figure S4.** Scatter plots for the causal association between gut microbiota and asthma-related pneumonia or sepsis.

**Additional file 12: Figure S5.** Leave-one-out plots for the causal association between gut microbiota and 28-day survival outcomes for sepsis.

**Additional file 13: Figure S6.** Leave-one-out plots for the causal association between gut microbiota and COPD/ asthma/ ILD-related pneumonia or pneumonia-derived septicaemia.

**Additional file 14: Figure S7.** Leave-one-out plots for the causal association between gut microbiota and COPD/ asthma-related pneumonia or pneumonia-derived septicaemia.

**Additional file 15: Figure S8.** Leave-one-out plots for the causal association between gut microbiota and asthma-related pneumonia or sepsis.

**Additional file 16: Figure S9.** Expression of potential key genes of COPD/ asthma/ ILD-related pneumonia or pneumonia-derived septicaemia associated with gut microbiota in sepsis single-cell data. (A, C) Expression of potential key genes in each cell type of sepsis samples. (B, D) Expression of potential key genes in each cell type of health samples.

**Additional file 17: Figure S10.** Expression of potential key genes of COPD/ asthma-related pneumonia or pneumonia-derived septicaemia associated with gut microbiota in sepsis single-cell data. (A, C) Expression of potential key genes in each cell type of sepsis samples. (B, D) Expression of potential key genes in each cell type of health samples.

**Additional file 18: Figure S11.** Expression of potential key genes of asthma-related pneumonia or sepsis associated with gut microbiota in sepsis single-cell data. (A, C) Expression of potential key genes in each cell type of sepsis samples. (B, D) Expression of potential key genes in each cell type of health samples.

**Additional file 19: Figure S12.** Gene expression in healthy people and sepsis patients. (A-G) Expression of NTSR1 (A), BCL6 (B), ZDHHC19 (C), MGLL (D), ALPK1 (E), VAV2 (F), and SATB1 (G) in healthy people and sepsis patients. (H) Expression of FCHO1 in sepsis patients who survived at 28 days and sepsis patients who died at 28 days.

## Data Availability

The datasets presented in this study can be found in online repositories. The names of the repository/repositories and accession number(s) can be found in the article/Additional Material.

## References

[1] Li J, Shen L, Qian K (2023). Global, regional, and national incidence and mortality of neonatal sepsis and other neonatal infections, 1990–2019. Front Public Health.

[2] Fleischmann-Struzek C, Mellhammar L, Rose N, Cassini A, Rudd KE, Schlattmann P, Allegranzi B, Reinhart K (2020). Incidence and mortality of hospital- and ICU-treated sepsis: results from an updated and expanded systematic review and meta-analysis. Intensive Care Med.

[3] Yang WS, Kang HD, Jung SK, Lee YJ, Oh SH, Kim YJ, Sohn CH, Kim WY (2020). A mortality analysis of septic shock, vasoplegic shock and cryptic shock classified by the third international consensus definitions (Sepsis-3). Clin Respir J.

[4] Lee J, Levy MM (2019). Treatment of patients with severe sepsis and septic shock: current evidence-based practices. Rhode Island Med J (2013).

[5] Du B, Shen N, Tao Y, Sun S, Zhang F, Ren H, Cao Q, Mo X (2021). Analysis of gut microbiota alteration and application as an auxiliary prognostic marker for sepsis in children: a pilot study. Transl Pediatr.

[6] Assimakopoulos SF, Triantos C, Thomopoulos K, Fligou F, Maroulis I, Marangos M, Gogos CA (2018). Gut-origin sepsis in the critically ill patient: pathophysiology and treatment. Infection.

[7] Sun S, Wang D, Dong D, Xu L, Xie M, Wang Y, Ni T, Jiang W, Zhu X, Ning N, Sun Q, Zhao S, Li M, Chen P, Yu M, Li J, Chen E, Zhao B, Peng Y, Mao E (2023). Altered intestinal microbiome and metabolome correspond to the clinical outcome of sepsis. Crit Care (London, England).

[8] Han Y, Gong Z, Sun G, Xu J, Qi C, Sun W, Jiang H, Cao P, Ju H (2021). Dysbiosis of gut microbiota in patients with acute myocardial infarction. Front Microbiol.

[9] Shimizu K, Yamada T, Ogura H, Mohri T, Kiguchi T, Fujimi S, Asahara T, Yamada T, Ojima M, Ikeda M, Shimazu T (2018). Synbiotics modulate gut microbiota and reduce enteritis and ventilator-associated pneumonia in patients with sepsis: a randomized controlled trial. Crit Care (London, England).

[10] Liang H, Song H, Zhang X, Song G, Wang Y, Ding X, Duan X, Li L, Sun T, Kan Q (2022). Metformin attenuated sepsis-related liver injury by modulating gut microbiota. Emerg Microb Infect.

[11] Fang H, Fang M, Wang Y, Zhang H, Li J, Chen J, Wu Q, He L, Xu J, Deng J, Liu M, Deng Y, Chen C (2022). Indole-3-propionic acid as a potential therapeutic agent for sepsis-induced gut microbiota disturbance. Microbiol Spectr.

[12] Haak BW, Wiersinga WJ (2017). The role of the gut microbiota in sepsis. Lancet Gastroenterol Hepatol.

[13] Kullberg RFJ, Wiersinga WJ, Haak BW (2021). Gut microbiota and sepsis: from pathogenesis to novel treatments. Curr Opin Gastroenterol.

[14] Adelman MW, Woodworth MH, Langelier C, Busch LM, Kempker JA, Kraft CS, Martin GS (2020). The gut microbiome's role in the development, maintenance, and outcomes of sepsis. Crit Care (London, England).

[15] Nabizadeh E, Sadeghi J, Ahangarzadeh Rezaee M, Hasani A, Samadi Kafil H, Ghotaslou A, Kadkhoda H, Ghotaslou R (2023). Interaction between altered gut microbiota and sepsis: a hypothesis or an authentic fact?. J Intensive Care Med.

[16] Bassetti M, Bandera A, Gori A (2020). Therapeutic potential of the gut microbiota in the management of sepsis. Crit Care (London, England).

[17] Dickson RP (2016). The microbiome and critical illness. Lancet Respir Med.

[18] Huang XQ, Qiu JK, Wang CH, Pan L, Xu JK, Pan XH, Ji XB, Mao MJ (2020). Sepsis secondary to multifocal *Enterococcus faecium* infection: a case report. Medicine.

[19] Weith M, Beyer A (2023). The next step in Mendelian randomization. Elife.

[20] Bowden J, Holmes MV (2019). Meta-analysis and Mendelian randomization: a review. Res Synth Methods.

[21] Yazar S, Alquicira-Hernandez J, Wing K, Senabouth A, Gordon MG, Andersen S, Lu Q, Rowson A, Taylor TRP, Clarke L, Maccora K, Chen C, Cook AL, Ye CJ, Fairfax KA, Hewitt AW, Powell JE (2022). Single-cell eQTL mapping identifies cell type-specific genetic control of autoimmune disease. Science (New York, N.Y.).

[22] Papalexi E, Satija R (2018). Single-cell RNA sequencing to explore immune cell heterogeneity. Nat Rev Immunol.

[23] Wang T, Zhang X, Liu Z, Yao T, Zheng D, Gan J, Yu S, Li L, Chen P, Sun J (2021). Single-cell RNA sequencing reveals the sustained immune cell dysfunction in the pathogenesis of sepsis secondary to bacterial pneumonia. Genomics.

[24] Li X, Liao Z, Deng Z, Chen N, Zhao L (2021). Combining bulk and single-cell RNA-sequencing data to reveal gene expression pattern of chondrocytes in the osteoarthritic knee. Bioengineered.

[25] Skrivankova VW, Richmond RC, Woolf BAR, Yarmolinsky J, Davies NM, Swanson SA, VanderWeele TJ, Higgins JPT, Timpson NJ, Dimou N, Langenberg C, Golub RM, Loder EW, Gallo V, Tybjaerg-Hansen A, Davey Smith G, Egger M, Richards JB (2021). Strengthening the reporting of observational studies in epidemiology using Mendelian randomization: the STROBE-MR statement. JAMA.

[26] von Elm E, Altman DG, Egger M, Pocock SJ, Gøtzsche PC, Vandenbroucke JP, Initiative STROBE (2007). The strengthening the reporting of observational studies in epidemiology (STROBE) statement: guidelines for reporting observational studies. Lancet (London, England).

[27] Bowden J, Davey Smith G, Burgess S (2015). Mendelian randomization with invalid instruments: effect estimation and bias detection through Egger regression. Int J Epidemiol.

[28] Kurilshikov A, Medina-Gomez C, Bacigalupe R, Radjabzadeh D, Wang J, Demirkan A, Le Roy CI, Raygoza Garay JA, Finnicum CT, Liu X, Zhernakova DV, Bonder MJ, Hansen TH, Frost F, Rühlemann MC, Turpin W, Moon JY, Kim HN, Lüll K, Barkan E (2021). Large-scale association analyses identify host factors influencing human gut microbiome composition. Nat Genet.

[29] Bycroft C, Freeman C, Petkova D, Band G, Elliott LT, Sharp K, Motyer A, Vukcevic D, Delaneau O, O'Connell J, Cortes A, Welsh S, Young A, Effingham M, McVean G, Leslie S, Allen N, Donnelly P, Marchini J (2018). The UK Biobank resource with deep phenotyping and genomic data. Nature.

[30] Rautanen A, Mills TC, Gordon AC, Hutton P, Steffens M, Nuamah R, Chiche JD, Parks T, Chapman SJ, Davenport EE, Elliott KS, Bion J, Lichtner P, Meitinger T, Wienker TF, Caulfield MJ, Mein C, Bloos F, Bobek I, Cotogni P (2015). Genome-wide association study of survival from sepsis due to pneumonia: an observational cohort study. Lancet Respir Med.

[31] Hernandez-Beeftink T, Guillen-Guio B, Lorenzo-Salazar JM, Corrales A, Suarez-Pajes E, Feng R, Rubio-Rodríguez LA, Paynton ML, Cruz R, García-Laorden MI, Prieto-González M, Rodríguez-Pérez A, Carriedo D, Blanco J, Ambrós A, González-Higueras E, Espinosa E, Muriel A, Tamayo E, Martin MM (2022). A genome-wide association study of survival in patients with sepsis. Crit Care (London, England).

[32] Kurki MI, Karjalainen J, Palta P, Sipilä TP, Kristiansson K, Donner KM, Reeve MP, Laivuori H, Aavikko M, Kaunisto MA, Loukola A, Lahtela E, Mattsson H, Laiho P, Della Briotta Parolo P, Lehisto AA, Kanai M, Mars N, Rämö J, Kiiskinen T (2023). FinnGen provides genetic insights from a well-phenotyped isolated population. Nature.

[33] Sanna S, van Zuydam NR, Mahajan A, Kurilshikov A, Vich Vila A, Võsa U, Mujagic Z, Masclee AAM, Jonkers DMAE, Oosting M, Joosten LAB, Netea MG, Franke L, Zhernakova A, Fu J, Wijmenga C, McCarthy MI (2019). Causal relationships among the gut microbiome, short-chain fatty acids and metabolic diseases. Nat Genet.

[34] Burgess S, Small DS, Thompson SG (2017). A review of instrumental variable estimators for Mendelian randomization. Stat Methods Med Res.

[35] Burgess S, Thompson SG (2017). Interpreting findings from Mendelian randomization using the MR-Egger method. Eur J Epidemiol.

[36] Bowden J, Davey Smith G, Haycock PC, Burgess S (2016). Consistent estimation in Mendelian randomization with some invalid instruments using a weighted median estimator. Genet Epidemiol.

[37] Hartwig FP, Davey Smith G, Bowden J (2017). Robust inference in summary data Mendelian randomization via the zero modal pleiotropy assumption. Int J Epidemiol.

[38] Sanderson E, Spiller W, Bowden J (2021). Testing and correcting for weak and pleiotropic instruments in two-sample multivariable Mendelian randomization. Stat Med.

[39] Verbanck M, Chen CY, Neale B, Do R (2018). Detection of widespread horizontal pleiotropy in causal relationships inferred from Mendelian randomization between complex traits and diseases. Nat Genet.

[40] Guan M, Yan L, Li R, Xu Y, Chen D, Li S, Ma F, Zhang X (2022). Integration of leave-one-out method and real-time live cell reporter array system to assess the toxicity of mixtures. Environ Res.

[41] Hemani G, Zheng J, Elsworth B, Wade KH, Haberland V, Baird D, Laurin C, Burgess S, Bowden J, Langdon R, Tan VY, Yarmolinsky J, Shihab HA, Timpson NJ, Evans DM, Relton C, Martin RM, Davey Smith G, Gaunt TR, Haycock PC (2018). The MR-Base platform supports systematic causal inference across the human phenome. Elife.

[42] Oscanoa J, Sivapalan L, Gadaleta E, Dayem Ullah AZ, Lemoine NR, Chelala C (2020). SNPnexus: a web server for functional annotation of human genome sequence variation (2020 update). Nucleic Acids Res.

[43] Lloyd-Jones LR, Holloway A, McRae A, Yang J, Small K, Zhao J, Zeng B, Bakshi A, Metspalu A, Dermitzakis M, Gibson G, Spector T, Montgomery G, Esko T, Visscher PM, Powell JE (2017). The genetic architecture of gene expression in peripheral blood. Am J Hum Genet.

[44] Wu T, Hu E, Xu S, Chen M, Guo P, Dai Z, Feng T, Zhou L, Tang W, Zhan L, Fu X, Liu S, Bo X, Yu G (2021). clusterProfiler 4.0: a universal enrichment tool for interpreting omics data. Innovation (Cambridge (Mass.)).

[45] Qiu X, Li J, Bonenfant J, Jaroszewski L, Mittal A, Klein W, Godzik A, Nair MG (2021). Dynamic changes in human single-cell transcriptional signatures during fatal sepsis. J Leukoc Biol.

[46] Hao Y, Hao S, Andersen-Nissen E, Mauck WM, Zheng S, Butler A, Lee MJ, Wilk AJ, Darby C, Zager M, Hoffman P, Stoeckius M, Papalexi E, Mimitou EP, Jain J, Srivastava A, Stuart T, Fleming LM, Yeung B, Rogers AJ (2021). Integrated analysis of multimodal single-cell data. Cell.

[47] Aran D, Looney AP, Liu L, Wu E, Fong V, Hsu A, Chak S, Naikawadi RP, Wolters PJ, Abate AR, Butte AJ, Bhattacharya M (2019). Reference-based analysis of lung single-cell sequencing reveals a transitional profibrotic macrophage. Nat Immunol.

[48] Scicluna BP, Wiewel MA, van Vught LA, Hoogendijk AJ, Klarenbeek AM, Franitza M, Toliat MR, Nürnberg P, Horn J, Bonten MJ, Schultz MJ, Cremer OL, van der Poll T (2018). Molecular biomarker to assist in diagnosing abdominal sepsis upon ICU admission. Am J Respir Crit Care Med.

[49] Azcarate-Peril MA, Roach J, Marsh A, Chey WD, Sandborn WJ, Ritter AJ, Savaiano DA, Klaenhammer TR (2021). A double-blind, 377-subject randomized study identifies *Ruminococcus, Coprococcus, Christensenella*, and *Collinsella* as long-term potential key players in the modulation of the gut microbiome of lactose intolerant individuals by galacto-oligosaccharides. Gut microbes.

[50] Yang R, Shan S, Shi J, Li H, An N, Li S, Cui K, Guo H, Li Z (2023). *Coprococcus eutactus*, a potent probiotic, alleviates colitis via acetate-mediated IgA response and microbiota restoration. J Agric Food Chem.

[51] Xin X, Wang Q, Qing J, Song W, Gui Y, Li X, Li Y (2022). Th17 cells in primary Sjögren's syndrome negatively correlate with increased *Roseburia* and *Coprococcus*. Front Immunol.

[52] Shen Y, Xu J, Li Z, Huang Y, Yuan Y, Wang J, Zhang M, Hu S, Liang Y (2018). Analysis of gut microbiota diversity and auxiliary diagnosis as a biomarker in patients with schizophrenia: a cross-sectional study. Schizophr Res.

[53] Sorbara MT, Littmann ER, Fontana E, Moody TU, Kohout CE, Gjonbalaj M, Eaton V, Seok R, Leiner IM, Pamer EG (2020). Functional and genomic variation between human-derived isolates of Lachnospiraceae reveals inter- and intra-species diversity. Cell Host Microbe.

[54] Vacca M, Celano G, Calabrese FM, Portincasa P, Gobbetti M, De Angelis M (2020). The controversial role of human gut Lachnospiraceae. Microorganisms.

[55] Sun D, Bai R, Zhou W, Yao Z, Liu Y, Tang S, Ge X, Luo L, Luo C, Hu GF, Sheng J, Xu Z (2021). Angiogenin maintains gut microbe homeostasis by balancing α-Proteobacteria and Lachnospiraceae. Gut.

[56] Zhang H, Zhuo S, Song D, Wang L, Gu J, Ma J, Gu Y, Ji M, Chen M, Guo Y (2021). Icariin inhibits intestinal inflammation of DSS-induced colitis mice through modulating intestinal flora abundance and modulating p-p65/p65 molecule. Turk J Gastroenterol.

[57] Wong J, Piceno YM, DeSantis TZ, Pahl M, Andersen GL, Vaziri ND (2014). Expansion of urease- and uricase-containing, indole- and p-cresol-forming and contraction of short-chain fatty acid-producing intestinal microbiota in ESRD. Am J Nephrol.

[58] Li G, Lin J, Zhang C, Gao H, Lu H, Gao X, Zhu R, Li Z, Li M, Liu Z (2021). Microbiota metabolite butyrate constrains neutrophil functions and ameliorates mucosal inflammation in inflammatory bowel disease. Gut microbes.

[59] Salvi PS, Cowles RA (2021). Butyrate and the intestinal epithelium: modulation of proliferation and inflammation in homeostasis and disease. Cells.

[60] Lin H, Ma X, Yang X, Chen Q, Wen Z, Yang M, Fu J, Yin T, Lu G, Qi J, Han H, Yang Y (2022). Natural shikonin and acetyl-shikonin improve intestinal microbial and protein composition to alleviate colitis-associated colorectal cancer. Int Immunopharmacol.

[61] Mukherjee A, Lordan C, Ross RP, Cotter PD (2020). Gut microbes from the phylogenetically diverse genus *Eubacterium* and their various contributions to gut health. Gut microbes.

[62] Kamel O, Van Noten H, Argudín MA, Martiny D (2021). Butyricimonas faecihominis and Eubacterium callanderi mixed bloodstream infection after appendicular peritonitis. Anaerobe.

[63] Crost EH, Coletto E, Bell A, Juge N (2023). *Ruminococcus gnavus*: friend or foe for human health. FEMS Microbiol Rev.

[64] Hall AB, Yassour M, Sauk J, Garner A, Jiang X, Arthur T, Lagoudas GK, Vatanen T, Fornelos N, Wilson R, Bertha M, Cohen M, Garber J, Khalili H, Gevers D, Ananthakrishnan AN, Kugathasan S, Lander ES, Blainey P, Vlamakis H (2017). A novel *Ruminococcus gnavus* clade enriched in inflammatory bowel disease patients. Genome Med.

[65] Henke MT, Brown EM, Cassilly CD, Vlamakis H, Xavier RJ, Clardy J (2021). Capsular polysaccharide correlates with immune response to the human gut microbe *Ruminococcus gnavus*. Proc Natl Acad Sci USA.

[66] Wang L, Christophersen CT, Sorich MJ, Gerber JP, Angley MT, Conlon MA (2013). Increased abundance of *Sutterella* spp. and *Ruminococcus* torques in feces of children with autism spectrum disorder. Mol Autism.

[67] Ren M, Zhang H, Qi J, Hu A, Jiang Q, Hou Y, Feng Q, Ojo O, Wang X (2020). An almond-based low carbohydrate diet improves depression and glycometabolism in patients with type 2 diabetes through modulating gut microbiota and GLP-1: a randomized controlled trial. Nutrients.

[68] Zafar H, Saier MH (2021). Gut *Bacteroides* species in health and disease. Gut Microb.

[69] Arnold Z, Wiggins A, Santos RL, Breighner C (2020). *Bacteroides* bundle of joy: sepsis from a degenerating/necrotic fibroid. BMJ Case Rep.

[70] Tsai AP, Dong C, Lin PB, Messenger EJ, Casali BT, Moutinho M, Liu Y, Oblak AL, Lamb BT, Landreth GE, Bissel SJ, Nho K (2022). PLCG2 is associated with the inflammatory response and is induced by amyloid plaques in Alzheimer's disease. Genome Med.

[71] Sims R, van der Lee SJ, Naj AC, Bellenguez C, Badarinarayan N, Jakobsdottir J, Kunkle BW, Boland A, Raybould R, Bis JC, Martin ER, Grenier-Boley B, Heilmann-Heimbach S, Chouraki V, Kuzma AB, Sleegers K, Vronskaya M, Ruiz A, Graham RR, Olaso R (2017). Rare coding variants in PLCG2, ABI3, and TREM2 implicate microglial-mediated innate immunity in Alzheimer's disease. Nat Genet.

[72] Aderibigbe OM, Priel DL, Lee CC, Ombrello MJ, Prajapati VH, Liang MG, Lyons JJ, Kuhns DB, Cowen EW, Milner JD (2015). Distinct cutaneous manifestations and cold-induced leukocyte activation associated with PLCG2 mutations. JAMA Dermatol.

[73] Szymanski AM, Ombrello MJ (2018). Using genes to triangulate the pathophysiology of granulomatous autoinflammatory disease: NOD2, PLCG2 and LACC1. Int Immunol.

[74] Zhang H, Qi X, Wu J, Huang X, Zhang A, Chen S, Ding X, Chen S, Le S, Zou Y, Xu H, Ye P, Xia J (2019). BCL6 inhibitor FX1 attenuates inflammatory responses in murine sepsis through strengthening BCL6 binding affinity to downstream target gene promoters. Int Immunopharmacol.

[75] Sawant DV, Sehra S, Nguyen ET, Jadhav R, Englert K, Shinnakasu R, Hangoc G, Broxmeyer HE, Nakayama T, Perumal NB, Kaplan MH, Dent AL (2012). Bcl6 controls the Th2 inflammatory activity of regulatory T cells by repressing Gata3 function. J Immunol (Baltimore, Md.: 1950).

[76] Wang J, Chen L, Qiang P (2021). The role of IGF2BP2, an m6A reader gene, in human metabolic diseases and cancers. Cancer Cell Int.

[77] Wang JN, Wang F, Ke J, Li Z, Xu CH, Yang Q, Chen X, He XY, He Y, Suo XG, Li C, Yu JT, Jiang L, Ni WJ, Jin J, Liu MM, Shao W, Yang C, Gong Q, Chen HY (2022). Inhibition of *METTL3* attenuates renal injury and inflammation by alleviating *TAB3* m6A modifications via IGF2BP2-dependent mechanisms. Sci Transl Med.

[78] Wang J, Yuan X, Ding N (2021). IGF2BP2 knockdown inhibits LPS-induced pyroptosis in BEAS-2B cells by targeting caspase 4, a crucial molecule of the non-canonical pyroptosis pathway. Exp Ther Med.

[79] Zhang J, Song Y, Shi Q, Fu L (2021). Research progress on FASN and MGLL in the regulation of abnormal lipid metabolism and the relationship between tumor invasion and metastasis. Front Med.

[80] Dione N, Lacroix S, Taschler U, Deschênes T, Abolghasemi A, Leblanc N, Di Marzo V, Silvestri C (2020). *Mgll* knockout mouse resistance to diet-induced dysmetabolism is associated with altered gut microbiota. Cells.

[81] Wilkins HM, Swerdlow RH (2017). Amyloid precursor protein processing and bioenergetics. Brain Res Bull.

[82] Guo Y, Wang Q, Chen S, Xu C (2021). Functions of amyloid precursor protein in metabolic diseases. Metab Clin Exp.

[83] Wu H, Zhou Y, Wu H, Xu L, Yan Y, Tong X, Yan H (2021). CENPN acts as a novel biomarker that correlates with the malignant phenotypes of glioma cells. Front Genet.

[84] Mertsch S, Schlicht K, Melkonyan H, Schlatt S, Thanos S (2018). snRPN controls the ability of neurons to regenerate axons. Restor Neurol Neurosci.

[85] Niu J, Sun Y, Chen B, Zheng B, Jarugumilli GK, Walker SR, Hata AN, Mino-Kenudson M, Frank DA, Wu X (2019). Fatty acids and cancer-amplified ZDHHC19 promote STAT3 activation through *S*-palmitoylation. Nature.

[86] Liang S, Zhang X, Li J (2022). Zinc finger Asp-His-His-Cys palmitoyl -acyltransferase 19 accelerates tumor progression through wnt/β-catenin pathway and is upregulated by miR-940 in osteosarcoma. Bioengineered.

[87] Fan X, Fan J, Yang H, Zhao C, Niu W, Fang Z, Chen X (2021). Heterogeneity of subsets in glioblastoma mediated by Smad3 palmitoylation. Oncogenesis.

[88] Arens C, Bajwa SA, Koch C, Siegler BH, Schneck E, Hecker A, Weiterer S, Lichtenstern C, Weigand MA, Uhle F (2016). Sepsis-induced long-term immune paralysis–results of a descriptive, explorative study. Crit Care (London, England).

[89] Hu HR, Dong Z, Yi L, He XY, Zhang YL, Liu YL, Cui HJ (2015). Function and mechanism of neurotensin (NTS) and its receptor 1 (NTSR1) in occurrence and development of tumors. Zhongguo Zhong yao za zhi = Zhongguo zhongyao zazhi = China J Chin Mater Med.

[90] Ko AM, Tu HP, Ko YC (2022). Systematic review of the role of alpha-protein kinase 1 in cancer and cancer-related inflammatory diseases. Cancers.

[91] Liu W, Miao C, Zhang S, Liu Y, Niu X, Xi Y, Guo W, Chu J, Lin A, Liu H, Yang X, Chen X, Zhong C, Ma Y, Wang Y, Zhu S, Liu S, Tan W, Lin D, Wu C (2021). VAV2 is required for DNA repair and implicated in cancer radiotherapy resistance. Signal Transduct Target Ther.

[92] Tartare-Deckert S, Monthouel MN, Charvet C, Foucault I, Van Obberghen E, Bernard A, Altman A, Deckert M (2001). Vav2 activates c-fos serum response element and CD69 expression but negatively regulates nuclear factor of activated T cells and interleukin-2 gene activation in T lymphocyte. J Biol Chem.

[93] Wells CM, Bhavsar PJ, Evans IR, Vigorito E, Turner M, Tybulewicz V, Ridley AJ (2005). Vav1 and Vav2 play different roles in macrophage migration and cytoskeletal organization. Exp Cell Res.

[94] Ju L, Zhou Z, Jiang B, Lou Y, Guo X (2017). Autocrine VEGF and IL-8 promote migration via Src/Vav2/Rac1/PAK1 signaling in human umbilical vein endothelial cells. Cell Physiol Biochem.

[95] Zelenka T, Spilianakis C (2020). SATB1-mediated chromatin landscape in T cells. Nucleus (Austin, Tex.).

[96] Chaurio RA, Anadon CM, Lee Costich T, Payne KK, Biswas S, Harro CM, Moran C, Ortiz AC, Cortina C, Rigolizzo KE, Sprenger KB, Mine JA, Innamarato P, Mandal G, Powers JJ, Martin A, Wang Z, Mehta S, Perez BA, Li R (2022). TGF-β-mediated silencing of genomic organizer SATB1 promotes Tfh cell differentiation and formation of intra-tumoral tertiary lymphoid structures. Immunity.

[97] Sunkara KP, Gupta G, Hansbro PM, Dua K, Bebawy M (2018). Functional relevance of SATB1 in immune regulation and tumorigenesis. Biomed Pharmacother Biomedecine & pharmacotherapie.

[98] Łyszkiewicz M, Ziętara N, Frey L, Pannicke U, Stern M, Liu Y, Fan Y, Puchałka J, Hollizeck S, Somekh I, Rohlfs M, Yilmaz T, Ünal E, Karakukcu M, Patiroğlu T, Kellerer C, Karasu E, Sykora KW, Lev A, Simon A (2020). Human FCHO1 deficiency reveals role for clathrin-mediated endocytosis in development and function of T cells. Nat Commun.

[99] Calzoni E, Platt CD, Keles S, Kuehn HS, Beaussant-Cohen S, Zhang Y, Pazmandi J, Lanzi G, Pala F, Tahiat A, Artac H, Heredia RJ, Dmytrus J, Reisli I, Uygun V, Uygun D, Bingol A, Basaran E, Djenouhat K, Benhalla N (2019). F-BAR domain only protein 1 (FCHO1) deficiency is a novel cause of combined immune deficiency in human subjects. J Allergy Clin Immunol.

[100] Horby P, Lim WS, Emberson JR, Mafham M, Bell JL, Linsell L, Staplin N, Brightling C, Ustianowski A, Elmahi E, Prudon B, Green C, Felton T, Chadwick D, Rege K, Fegan C, Chappell LC, Faust SN, Jaki T, RECOVERY Collaborative Group (2021). Dexamethasone in hospitalized patients with covid-19. N Engl J Med.

[101] Son JY, Shin S, Choi YJ (2021). New evidence of potential benefits of dexamethasone and added on therapy of fludrocortisone on clinical outcomes of corticosteroid in sepsis patients: a systematic review and meta-analysis. J Personalized Med.

[102] Feng LH, Li XD, Zhang XY, Cheng PJ, Feng ZY (2022). Dexamethasone for the treatment of acute respiratory distress syndrome: a systematic review and meta-analysis. Medicine.

[103] Allou N, Bouteau A, Allyn J, Snauwaert A, Valance D, Jabot J, Bouchet B, Galliot R, Corradi L, Montravers P, Augustin P (2016). Impact of a high loading dose of amikacin in patients with severe sepsis or septic shock. Ann Intensive Care.

[104] Li J, Zeng X, Yang F, Wang L, Luo X, Liu R, Zeng F, Lu S, Huang X, Lei Y, Lan Y (2022). Resveratrol: potential application in sepsis. Front Pharmacol.

