# Supplementary figures and images for: Causal effects of gut microbiota on sepsis and sepsis-related death: insights from genome-wide Mendelian randomization, single-cell RNA, bulk RNA sequencing, and network pharmacology
Source: J Transl Med. 2024 Jan 2;22:10. doi: 10.1186/s12967-023-04835-8 (PMC10763396; doi:10.1186/s12967-023-04835-8)

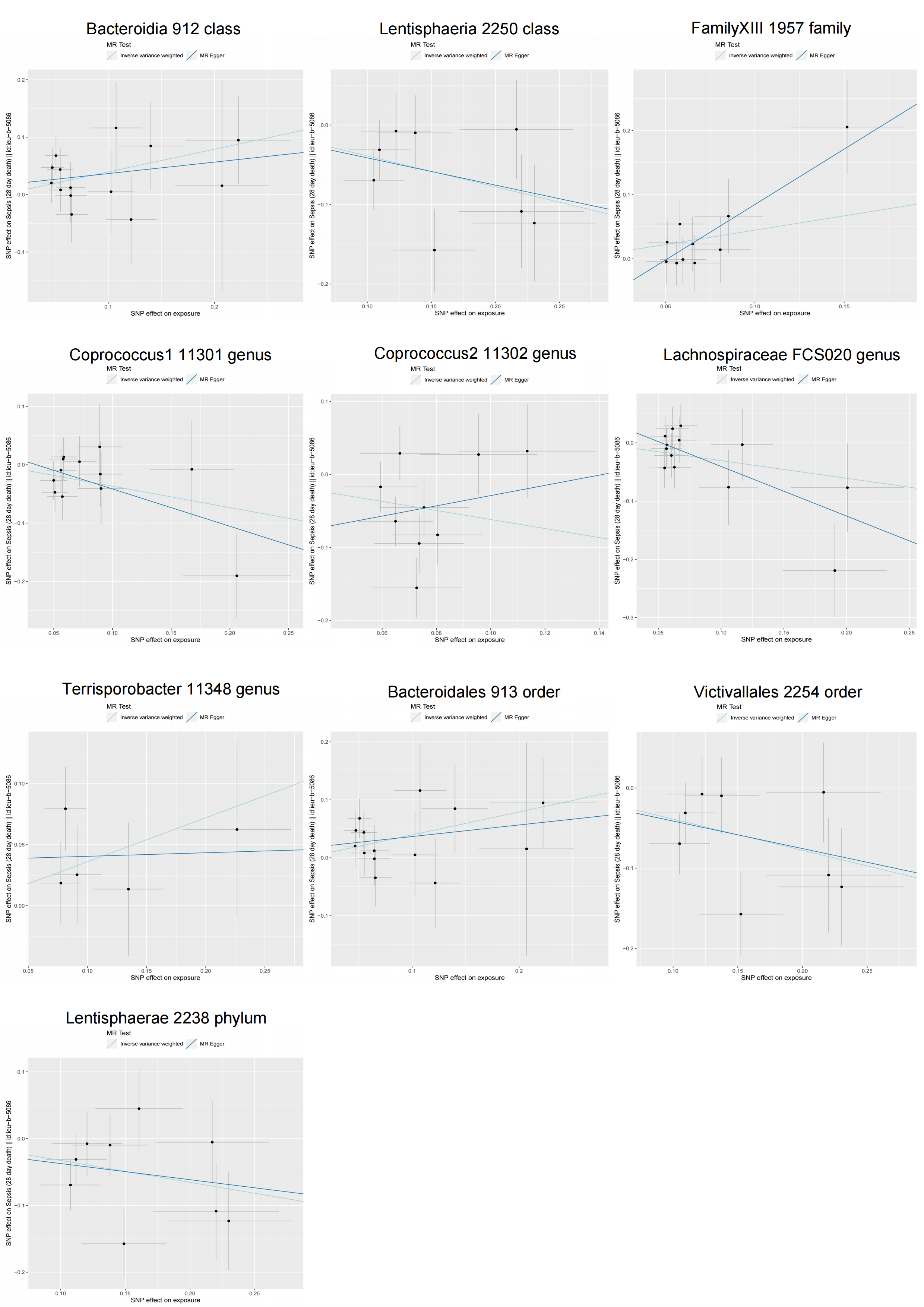

Supplement: Supplementary file 8 — Additional file 8: Figure S1. Scatter plots for the causal association between gut microbiota and 28-day survival outcomes for sepsis. [file 12967_2023_4835_MOESM8_ESM.tif]

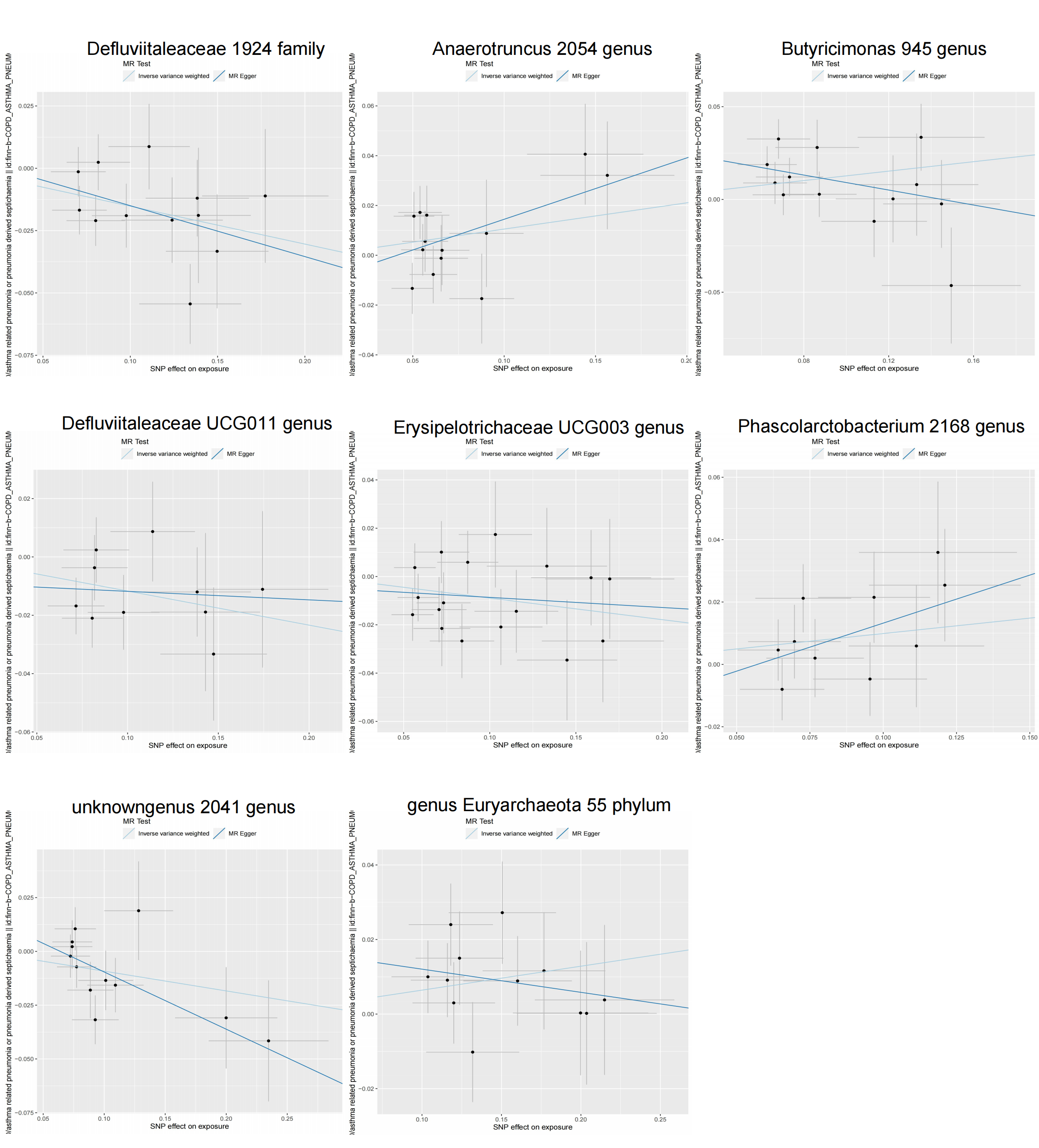

Supplement: Supplementary file 9 — Additional file 9: Figure S2. Scatter plots for the causal association between gut microbiota and COPD/ asthma/ ILD-related pneumonia or pneumonia-derived septicaemia. [file 12967_2023_4835_MOESM9_ESM.tif]

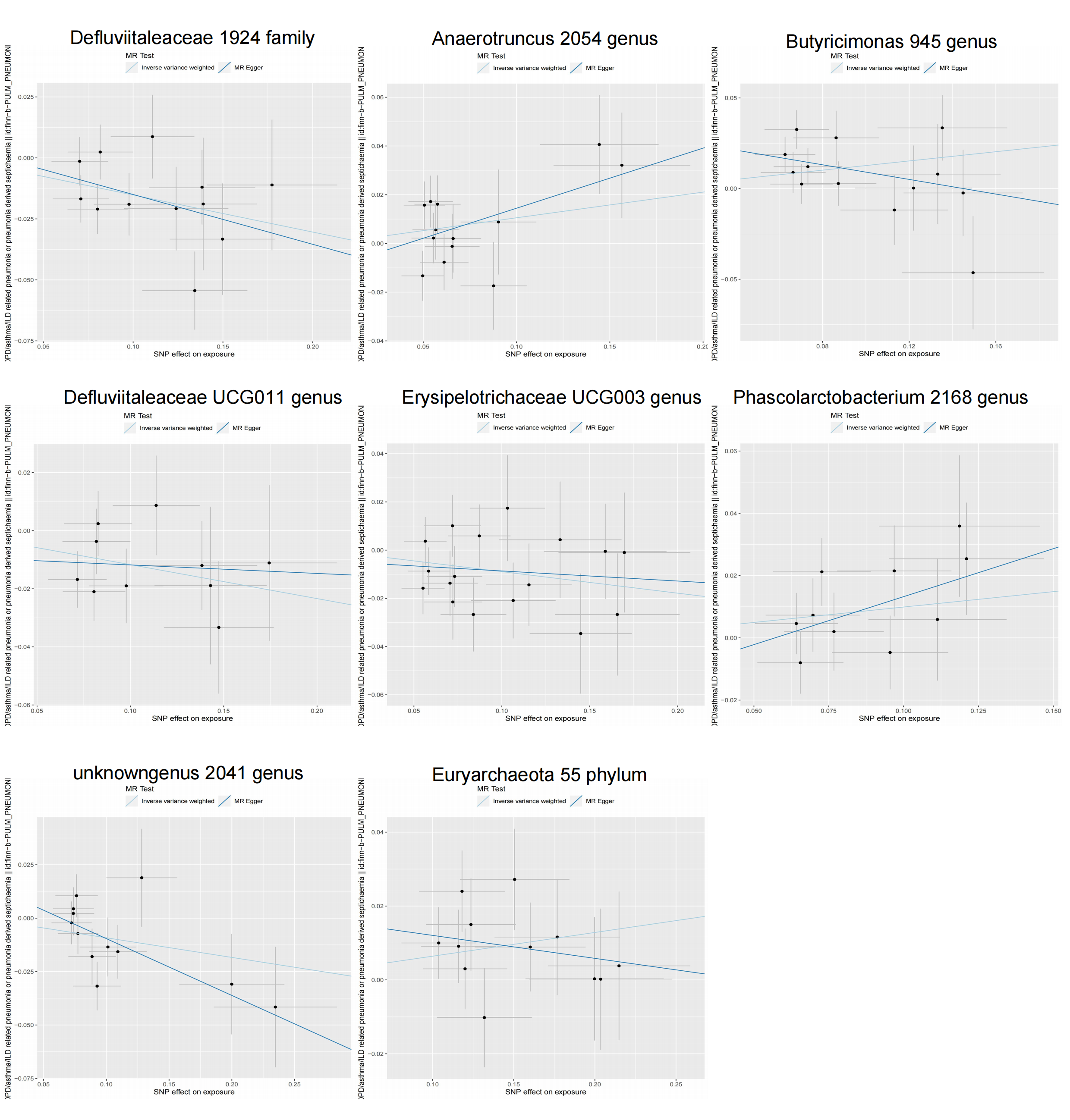

Supplement: Supplementary file 10 — Additional file 10: Figure S3. Scatter plots for the causal association between gut microbiota and COPD/ asthma-related pneumonia or pneumonia-derived septicaemia. [file 12967_2023_4835_MOESM10_ESM.tif]

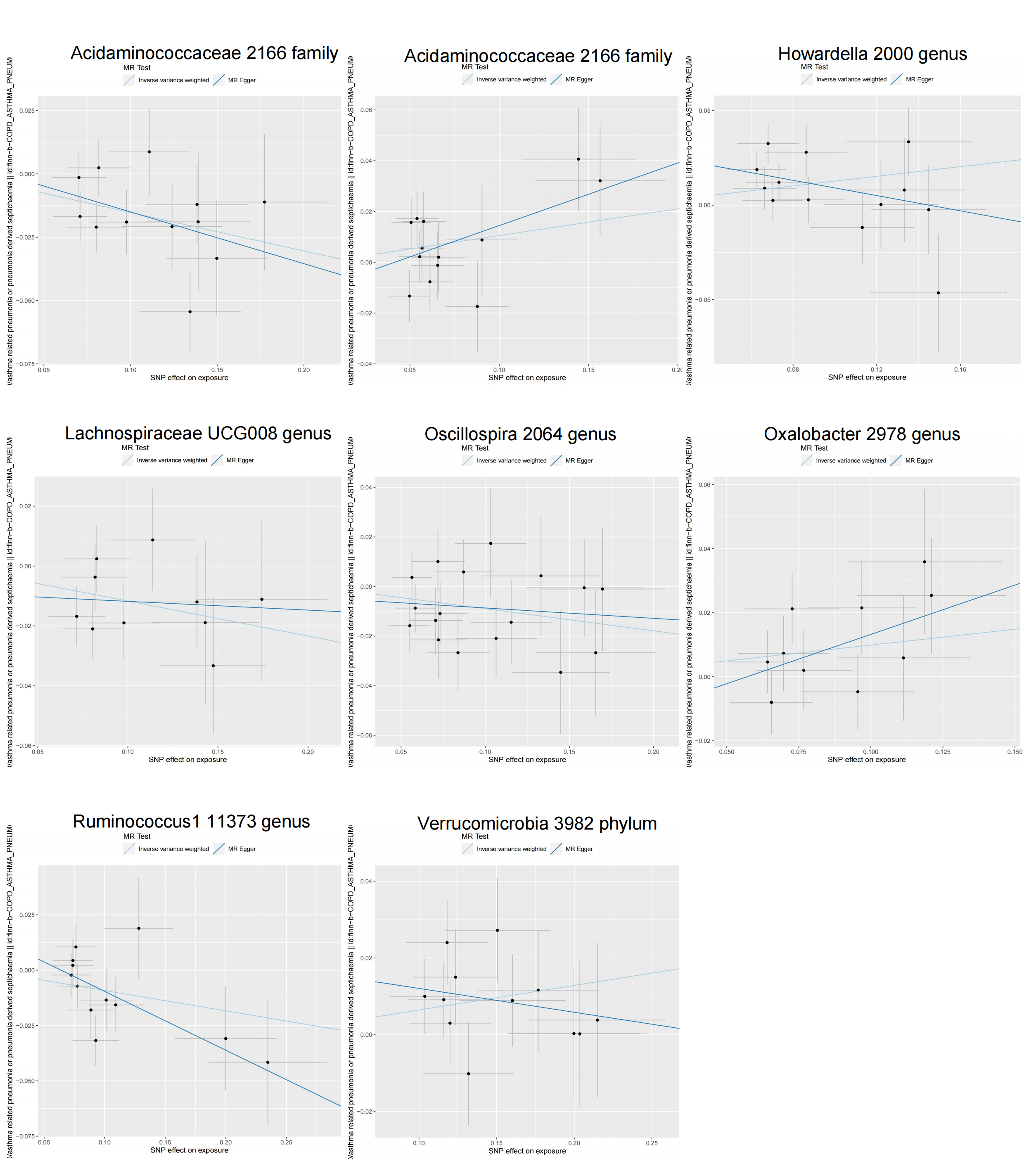

Supplement: Supplementary file 11 — Additional file 11: Figure S4. Scatter plots for the causal association between gut microbiota and asthma-related pneumonia or sepsis. [file 12967_2023_4835_MOESM11_ESM.tif]

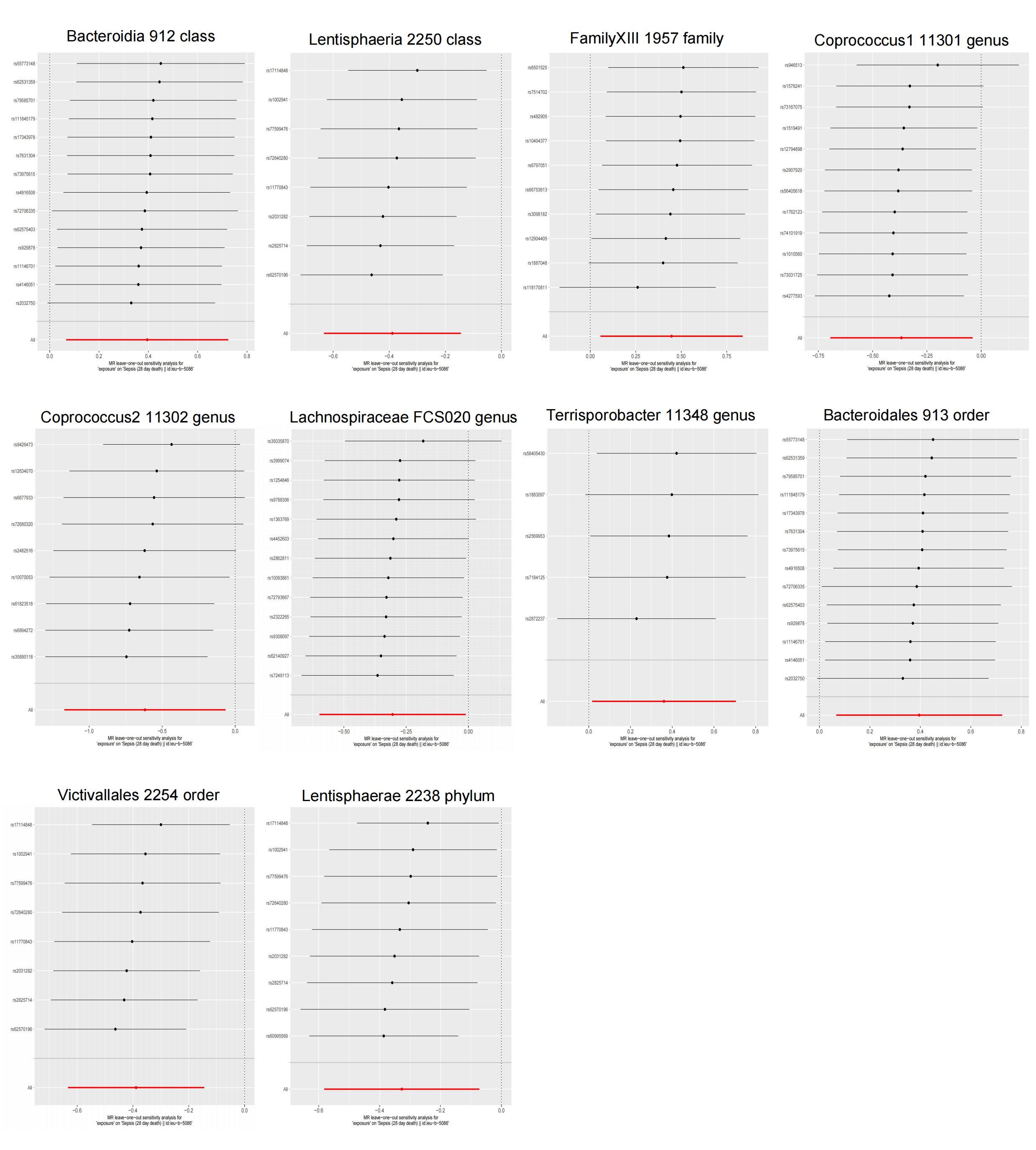

Supplement: Supplementary file 12 — Additional file 12: Figure S5. Leave-one-out plots for the causal association between gut microbiota and 28-day survival outcomes for sepsis. [file 12967_2023_4835_MOESM12_ESM.tif]

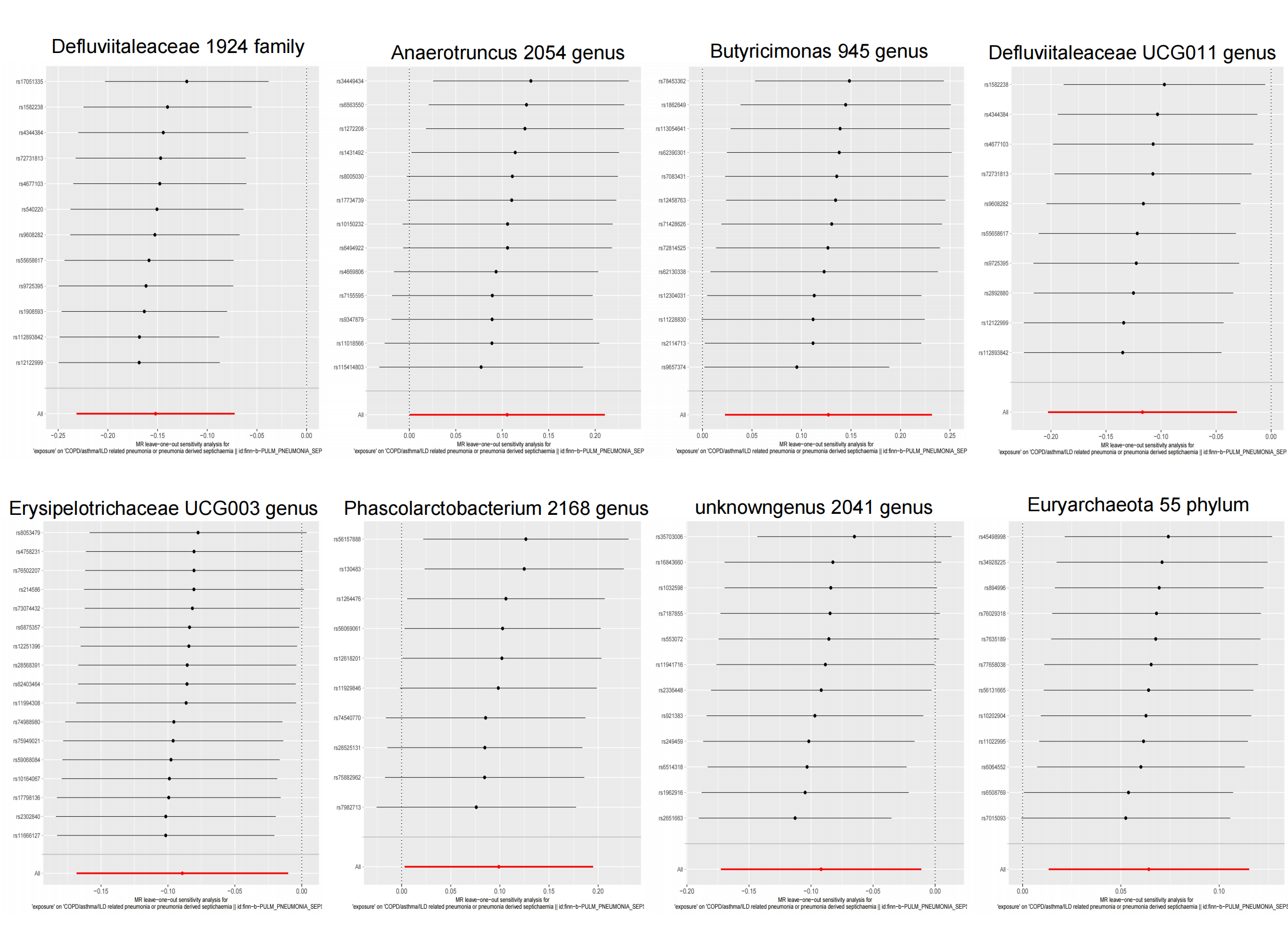

Supplement: Supplementary file 13 — Additional file 13: Figure S6. Leave-one-out plots for the causal association between gut microbiota and COPD/ asthma/ ILD-related pneumonia or pneumonia-derived septicaemia. [file 12967_2023_4835_MOESM13_ESM.tif]

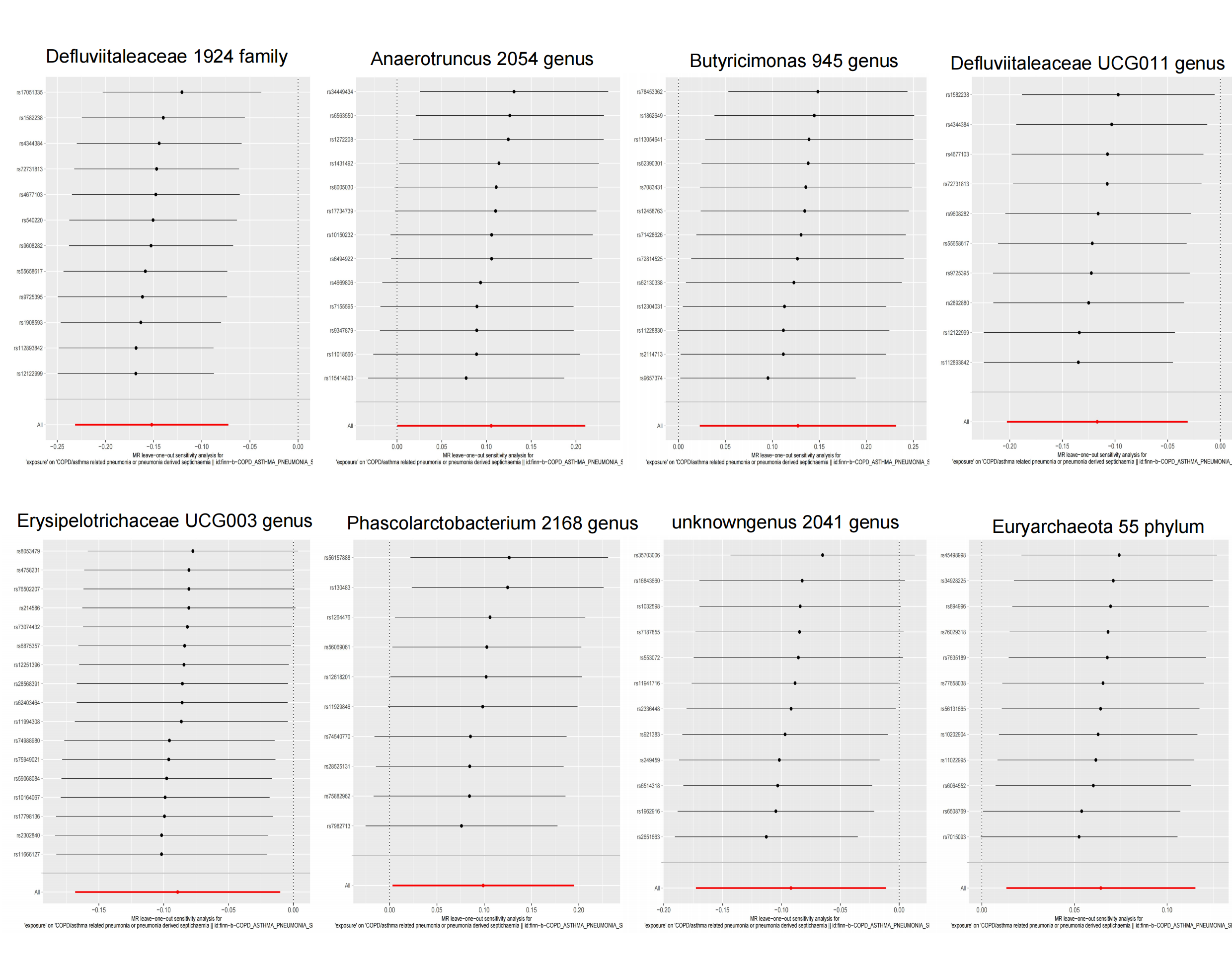

Supplement: Supplementary file 14 — Additional file 14: Figure S7. Leave-one-out plots for the causal association between gut microbiota and COPD/ asthma-related pneumonia or pneumonia-derived septicaemia. [file 12967_2023_4835_MOESM14_ESM.tif]

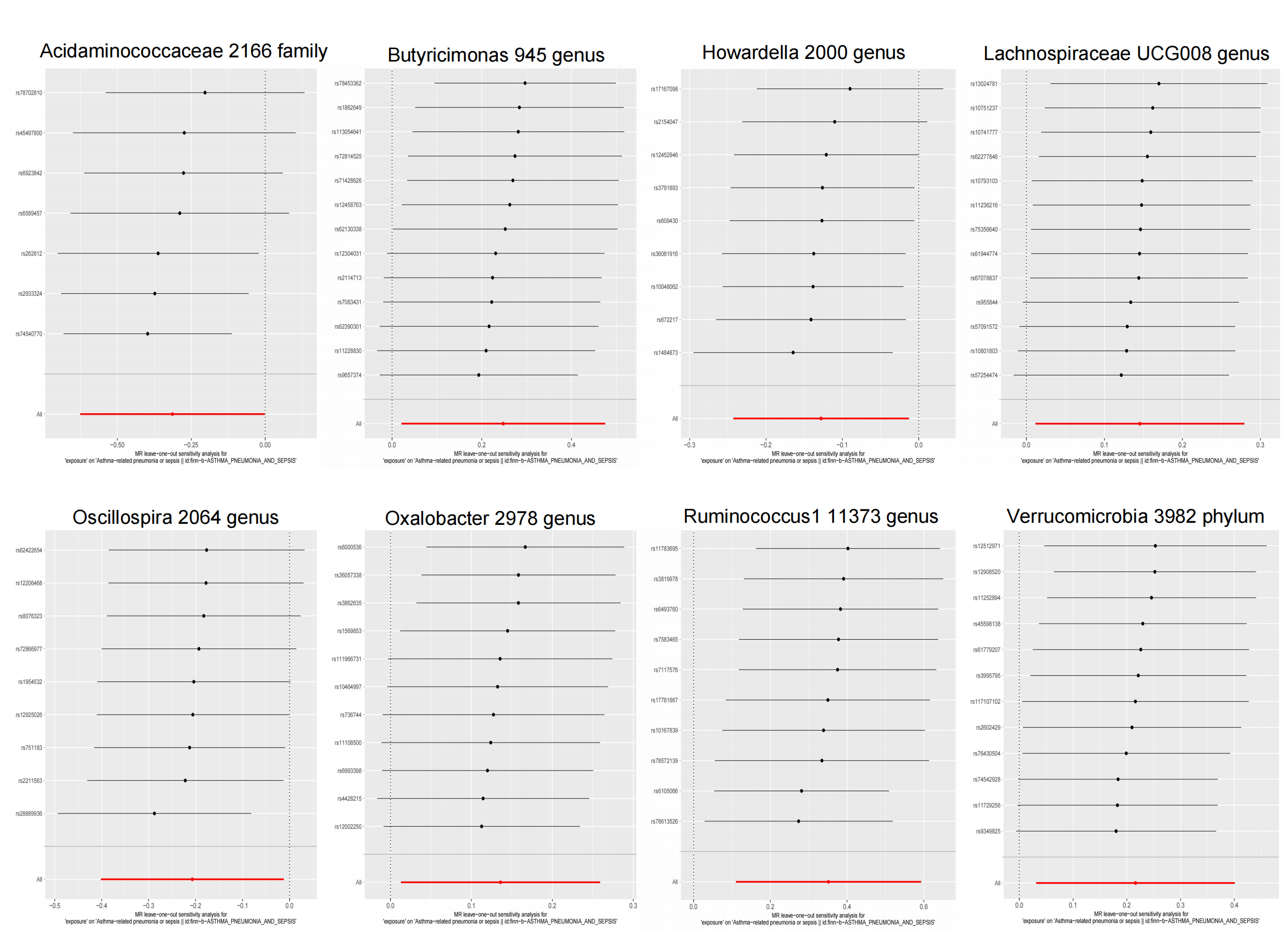

Supplement: Supplementary file 15 — Additional file 15: Figure S8. Leave-one-out plots for the causal association between gut microbiota and asthma-related pneumonia or sepsis. [file 12967_2023_4835_MOESM15_ESM.tif]

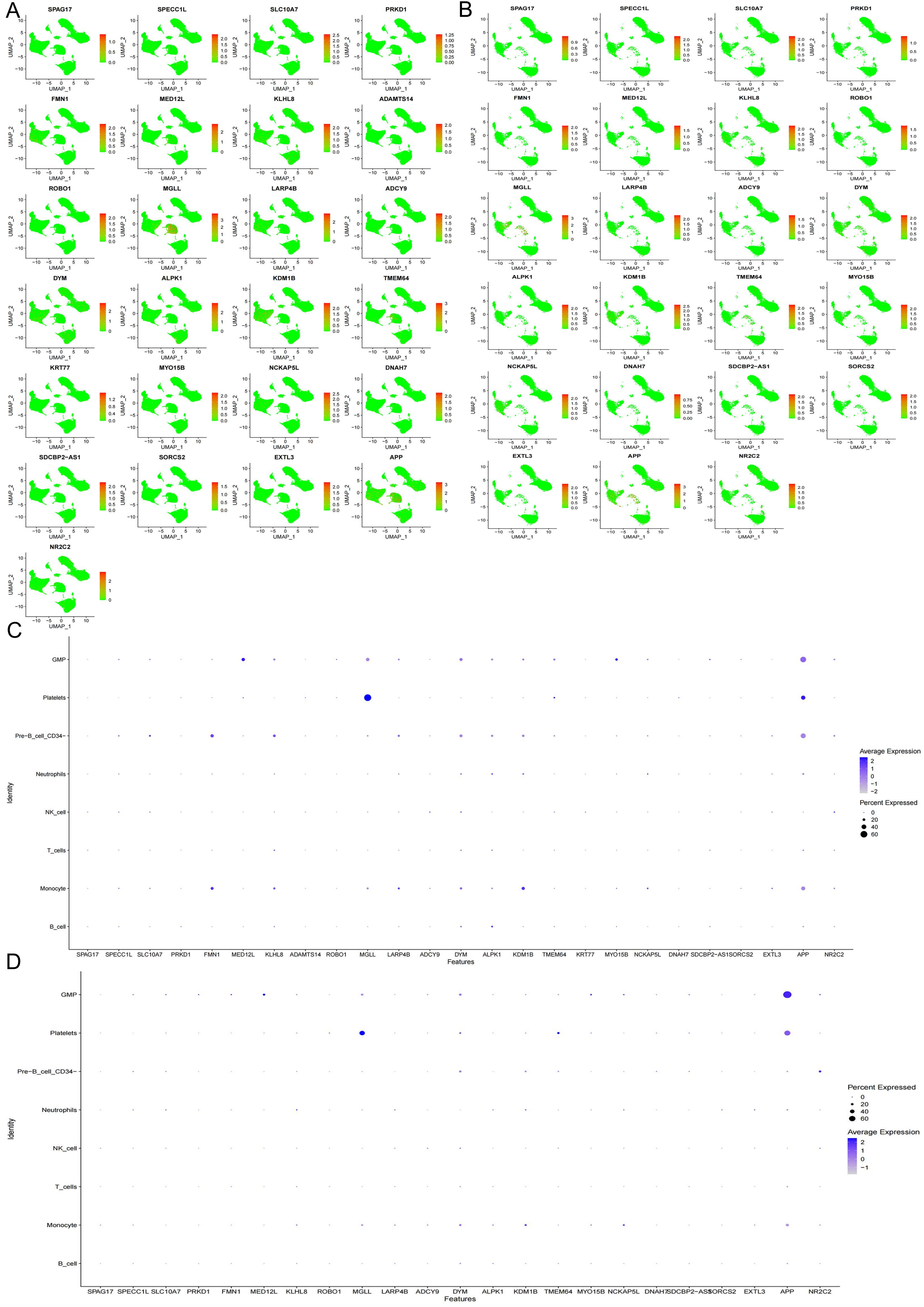

Supplement: Supplementary file 16 — Additional file 16: Figure S9. Expression of potential key genes of COPD/ asthma/ ILD-related pneumonia or pneumonia-derived septicaemia associated with gut microbiota in sepsis single-cell data. (A, C) Expression of potential key genes in each cell type of sepsis samples. (B, D) Expression of potential key genes in each cell type of health samples. [file 12967_2023_4835_MOESM16_ESM.tif]

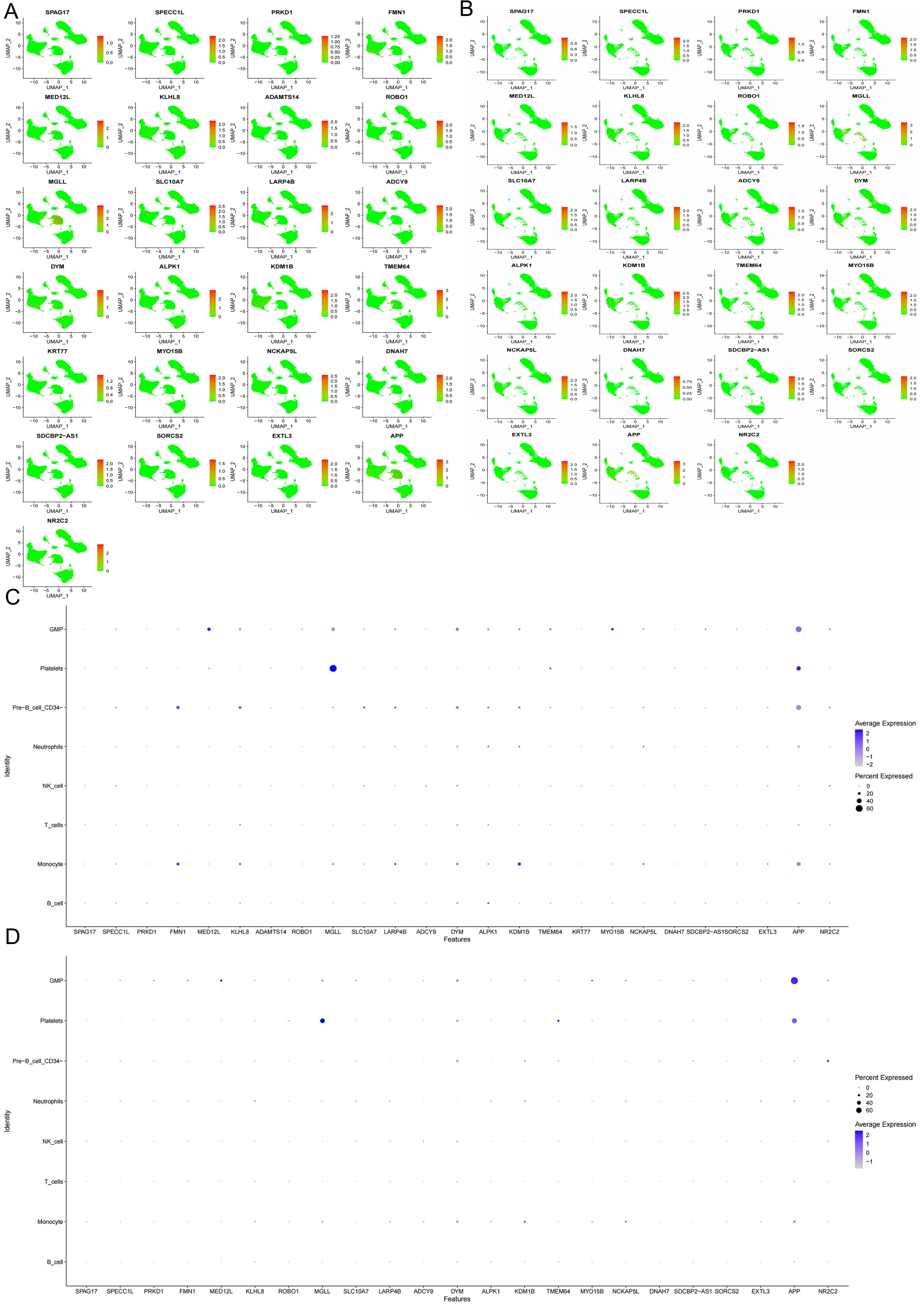

Supplement: Supplementary file 17 — Additional file 17: Figure S10. Expression of potential key genes of COPD/ asthma-related pneumonia or pneumonia-derived septicaemia associated with gut microbiota in sepsis single-cell data. (A, C) Expression of potential key genes in each cell type of sepsis samples. (B, D) Expression of potential key genes in each cell type of health samples. [file 12967_2023_4835_MOESM17_ESM.tif]

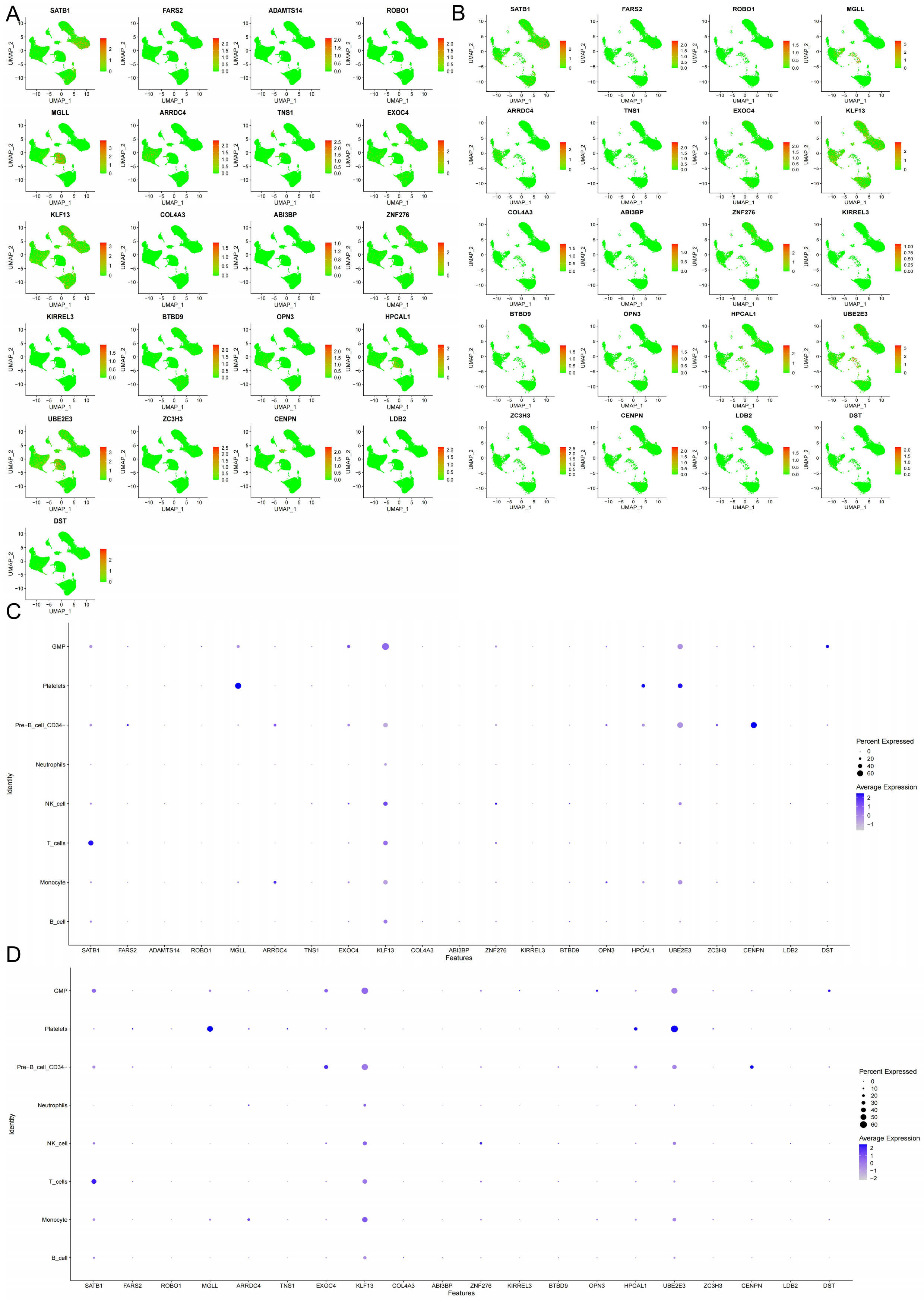

Supplement: Supplementary file 18 — Additional file 18: Figure S11. Expression of potential key genes of asthma-related pneumonia or sepsis associated with gut microbiota in sepsis single-cell data. (A, C) Expression of potential key genes in each cell type of sepsis samples. (B, D) Expression of potential key genes in each cell type of health samples. [file 12967_2023_4835_MOESM18_ESM.tif]

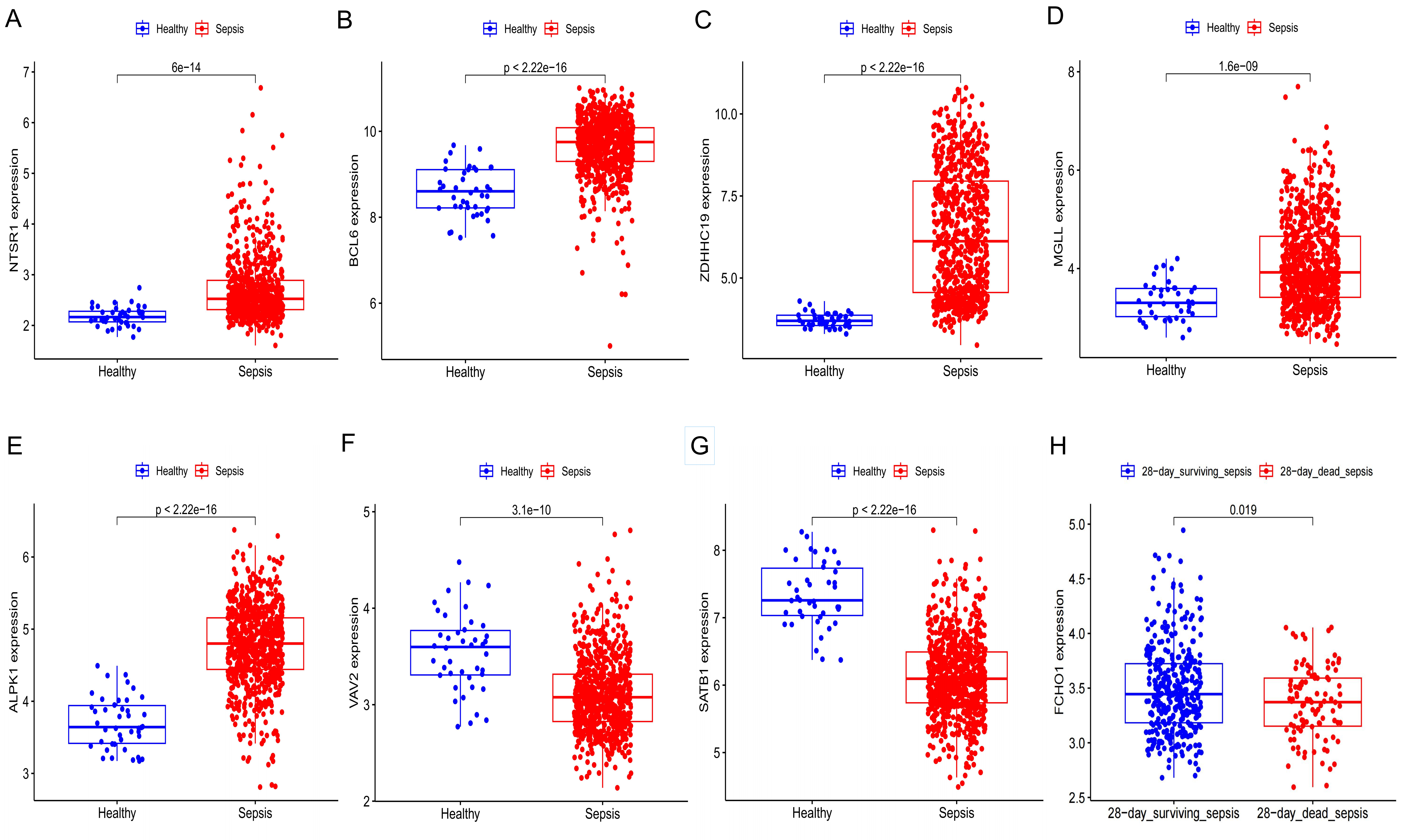

Supplement: Supplementary file 19 — Additional file 19: Figure S12. Gene expression in healthy people and sepsis patients. (A-G) Expression of NTSR1 (A), BCL6 (B), ZDHHC19 (C), MGLL (D), ALPK1 (E), VAV2 (F), and SATB1 (G) in healthy people and sepsis patients. (H) Expression of FCHO1 in sepsis patients who survived at 28 days and sepsis patients who died at 28 days. [file 12967_2023_4835_MOESM19_ESM.tif]
